# Robust Range-Doppler Acoustic Feature Fusion for Unauthorised Personnel Crossing Detection on Underground Belt Conveyors

**DOI:** 10.3390/s26196296

**Published:** 2026-10-05

**Authors:** Yuqing Yin, Baoxuan Xu, Xu Yang, Wenhao Guo

**Affiliations:** 1 School of Computer Science and Technolgy/School of Artifficial Intelligence, China University of Mining and Technology, Xuzhou 221116, China; yinyuqing@cumt.edu.cn (Y.Y.); xubaoxuan@cumt.edu.cn (B.X.); 2 State Key Laboratory of Digital Intelligent Technology for Unmanned Coal Mining, Huainan 232001, China; 3 School of Geology and Mining Engineering, Xinjiang University, Urumqi 830046, China

**Keywords:** belt conveyor, personnel crossing detection, acoustic sensing, distance–velocity fusion, temporal discrimination model

## Abstract

Unauthorised personnel crossing underground belt conveyors poses a serious safety risk in coal mines. To improve the reliability of non-contact crossing detection under complex acoustic interference, this paper proposes an acoustic sensing method based on distance–velocity feature fusion. An adaptive acoustic interference suppression and echo enhancement method is first employed to mitigate multipath interference and suppress mechanical vibration and background noise. Time-of-flight (ToF) and Doppler information are then jointly extracted from FMCW acoustic echoes to obtain the temporal distance trajectory and radial velocity of human targets. Based on these complementary features, a distance–velocity coupled temporal discrimination model is developed to distinguish continuous human crossing behavior from static obstacles and transient mechanical interference through spatial constraints, motion verification, and temporal state transitions. Experiments conducted on a simulated conveyor belt platform demonstrate that the proposed method achieves a detection accuracy of 96.5%, outperforming single-feature acoustic detection methods and maintaining stable detection performance under different sensing distances, motion directions, noise levels, and conveyor operating conditions.

## 1. Introduction

Underground belt conveyors are essential for coal transportation, connecting mining faces with surface facilities. Illegal crossing of operating conveyor belts is a major cause of underground accidents, often resulting in severe injuries and production losses [1]. However, harsh underground conditions and continuous equipment operation limit the effectiveness of traditional protective measures [2]. Therefore, reliable intelligent detection methods for conveyor belt safety monitoring are urgently needed in intelligent mines [3].

Existing underground personnel detection technologies can be mainly classified into four categories: wearable positioning, vision-based sensing, infrared barriers, and millimeter-wave radar [4]. Wearable positioning systems rely on personnel identification devices and electronic fences, but suffer from problems such as missing tags, signal obstruction, and insufficient localization accuracy, making them ineffective for detecting dynamic illegal crossing behaviors [5]. Vision-based approaches perform well under favorable illumination conditions but are vulnerable to image degradation caused by underground dust, water mist, and low-light environments [6]. Infrared barrier systems are easily affected by dust and moisture attenuation, while falling debris may trigger false alarms [7]. Millimeter-wave radar provides contactless distance and velocity measurements, but its high cost and susceptibility to electromagnetic interference limit large-scale deployment in underground mines [8].

Wireless non-contact sensing technologies, such as WiFi, radar, and acoustic sensing, have attracted increasing attention in mine safety monitoring [9]. Acoustic sensing is particularly suitable for underground environments due to its independence from illumination conditions, its comparatively lower sensitivity to airborne dust than vision-based and infrared sensing, and its low deployment cost [10]. However, existing acoustic studies mainly focus on equipment monitoring and environmental perception, relying on single features such as amplitude or energy. The lack of distance and motion information limits their ability to distinguish dynamic human targets from static obstacles, leading to high false alarm rates [11].

To address these limitations, this study proposes a conveyor belt personnel violation detection method based on acoustic echo distance–velocity dual-feature fusion [12]. A general-purpose acoustic sensing system is employed to achieve low-cost and illumination-independent non-contact monitoring. Three major challenges are addressed: (1) acoustic signals in confined underground tunnels are severely affected by multipath reflections, mechanical vibrations, and airflow disturbances, resulting in low signal-to-noise ratios and weak human echo responses; (2) conventional acoustic detection methods lack coupled distance and velocity information, making it difficult to distinguish moving personnel from static objects; and (3) personnel crossing behaviors exhibit continuous motion patterns, including approaching, crossing, and leaving, which cannot be accurately identified using single-threshold detection [13,14].

**Figure 1:**
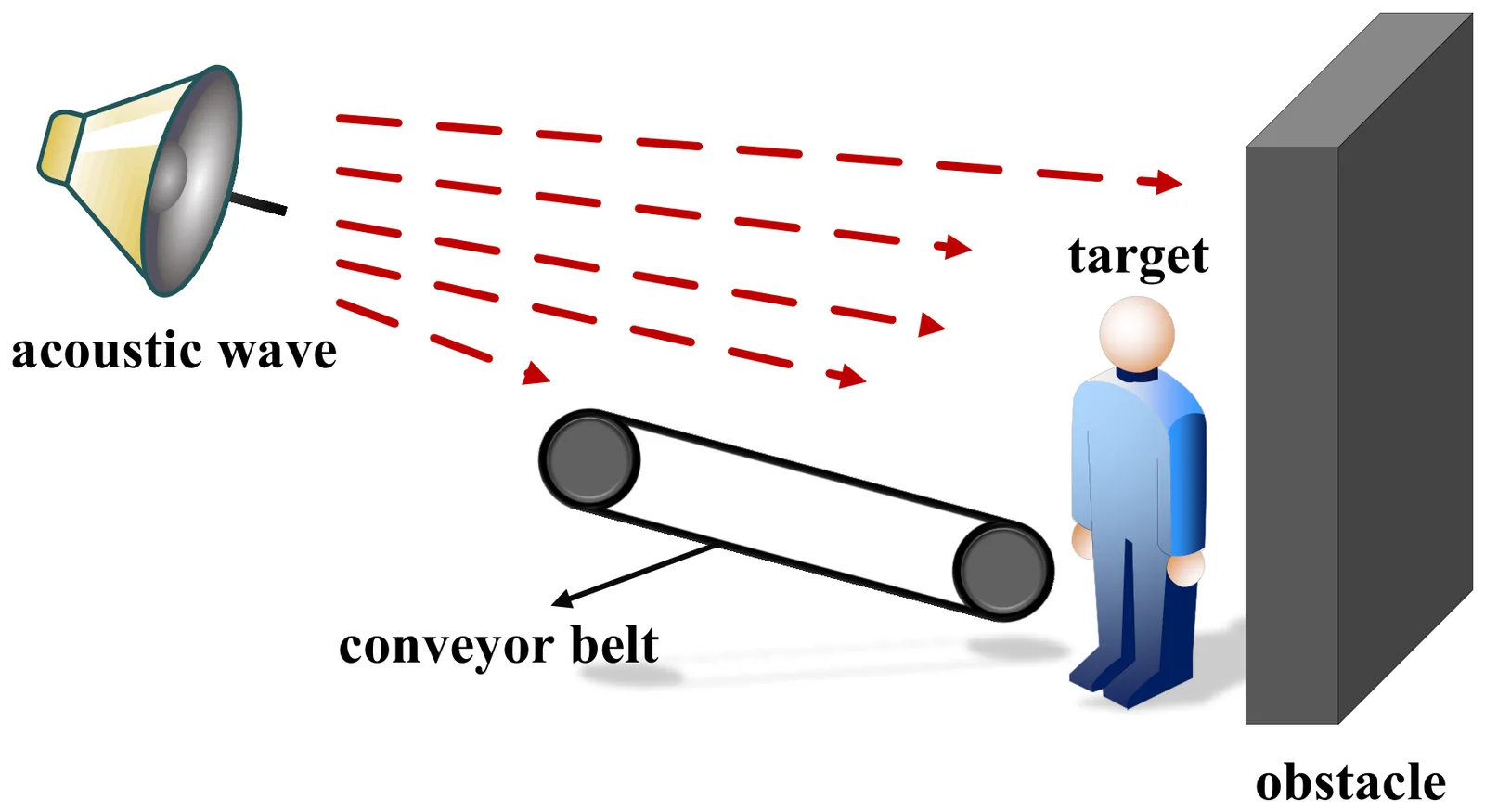
Illustration of the FMCW acoustic sensing scenario in underground environments. The transmitted acoustic wave propagates through the tunnel and interacts with human targets, conveyor belts, and surrounding obstacles, resulting in complex reflected echoes caused by multipath propagation.

To overcome these challenges, an adaptive multi-stage filtering algorithm is developed to suppress multipath interference and vibration noise while enhancing human target echoes. Time-of-flight (TOF) and Doppler-based methods are further integrated to simultaneously extract temporal distance and radial velocity features. Based on these features, a distance-constrained and velocity-validated fusion model with temporal continuity constraints is constructed, enabling reliable discrimination between human crossing behaviors and environmental disturbances. The overall sensing scenario of the Frequency-Modulated Continuous Wave (FMCW) acoustic system is illustrated in Figure 1.

The main contributions of this study are summarized as follows:

- An acoustic echo-based distance–velocity dual-feature fusion framework is proposed for unauthorized personnel crossing detection around underground conveyor belts, providing a non-visual sensing approach for continuous human behavior monitoring;
- An adaptive acoustic interference suppression and echo enhancement method is developed to mitigate multipath interference, mechanical vibration, and background noise. Based on the complementary distance and velocity features, a temporal-constrained dual-feature decision model is further constructed to improve detection robustness and reduce false alarms;
- Extensive laboratory experiments are conducted on a simulated conveyor belt platform under different sensing distances, motion directions, acoustic noise levels, and conveyor operating conditions. Comparative and ablation experiments demonstrate the effectiveness and robustness of the proposed distance–velocity fusion method under controlled laboratory conditions.

The remainder of this paper is organized as follows. Section 2 reviews related studies on conveyor safety monitoring, underground acoustic sensing, and multi-feature fusion algorithms. Section 3 introduces the acoustic propagation characteristics in confined tunnels, echo differences between human targets and obstacles, and the principles of TOF and Doppler measurements. Section 4 describes the acoustic sensing hardware architecture, signal acquisition process, and adaptive denoising algorithm. Section 5 presents the distance and velocity feature extraction methods and the proposed fusion-based recognition model. Section 6 evaluates the proposed approach through comparative experiments under various operating conditions. Finally, Section 7 concludes this paper and discusses future research directions.

## 2. Related Work

### 2.1. Existing Personnel Detection Technologies

Existing personnel detection methods for underground conveyor belt hazardous areas mainly include wearable positioning, optical sensing, and radar-based non-contact sensing. These approaches provide different solutions for personnel monitoring but still face challenges under complex underground conditions.

Wearable monitoring systems identify personnel locations by collecting information from wearable tags or terminals and establishing electronic fences. Jiang et al. [15] developed an intrusion warning model based on a pseudolite positioning system by integrating personnel coordinates with conveyor hazard boundaries. Cavur and Demir [16] proposed an RSSI-based hybrid real-time tracking approach using RFID technology for underground mining environments. Fang et al. [17] developed and optimized a real-time localization system based on ultra-wideband (UWB) technology for personnel positioning in underground mines. However, wearable approaches mainly provide position information and cannot effectively distinguish behaviors such as standing, walking, and crossing. Signal blockage, missing tags, and limited battery capacity further restrict their application in dynamic violation detection.

Optical-based monitoring methods, including visible-light vision and infrared thermal imaging, are widely applied in mine safety monitoring. Ren et al. [18] developed a lightweight YOLOv5-based method for real-time safety monitoring of mining conveyor belts, enabling the detection of lump coal and belt surface damage. Ni et al. [19] introduced a coordinate attention mechanism into YOLOv8 and combined it with a ray-based method to detect personnel entering hazardous underground areas. Liu et al. [20] combined YOLOv7 with DeepSORT to achieve object detection and trajectory tracking. Nevertheless, dust, water mist, and poor illumination can significantly degrade the performance of optical sensing systems. In addition, explosion-proof cameras and edge computing devices may increase deployment costs. Imam et al. [21] reviewed computer-vision-based anti-collision systems for pedestrian detection in underground mines and highlighted the challenges associated with limited visibility and complex underground environments. Wei et al. [22] proposed a shearer-onboard personnel detection method based on infrared thermal imaging to address the limitations of visible-light imaging under poor illumination conditions in underground mining faces.

Millimeter-wave radar and ultra-wideband (UWB) radar provide contactless detection by measuring target distance and velocity. Zhai et al. developed a human motion detection system based on a single-chip millimeter-wave radar and extracted body motion features through modal decomposition. However, underground environments contain various electrical devices that introduce electromagnetic interference, and the high cost of explosion-proof radar limits large-scale deployment. Therefore, robust and low-cost sensing technologies with strong environmental adaptability remain highly desirable for underground personnel monitoring.

### 2.2. Acoustic Sensing in Mining and General Scenarios

Acoustic sensing has attracted increasing attention in underground mining due to its advantages of illumination independence, dust resistance, and low hardware cost. Existing studies mainly focus on geological monitoring, equipment diagnosis, and disaster warning, whereas acoustic-based human dynamic behavior recognition remains insufficiently explored.

For geological and disaster monitoring, Chambers et al. [23] investigated distributed acoustic sensing (DAS) for longwall coal mines and demonstrated multiple fiber-optic sensing deployment strategies for monitoring underground mining environments. Huang et al. [24] analyzed acoustic emission characteristics associated with damage evolution and deformation stability in mining slopes. Sun et al. [25] investigated a sound-recognition-based alarm method for major coal mine accidents. Cao et al. [26] applied DAS technology to monitor and identify overburden fracture signals in coal mine goaf areas. However, these methods primarily focus on recognizing acoustic events or monitoring structural vibration and fracture signals, rather than explicitly estimating the distance and velocity of moving human targets. Therefore, their direct application to dynamic personnel intrusion and crossing detection remains limited.

Acoustic sensing has also been applied to equipment condition monitoring. Gabor et al. [27] proposed a bearing damage detection method based on orthogonal and non-negative low-rank feature extaction, and validated its effectiveness using acoustic signals collected from a belt conveyor. Personnel localization is another important component of underground mine safety monitoring. Capraro et al. [28] developed a real-time localization system based on ultra-wideband (UWB) technology for tracking personnel and assets in underground mines. Although these approaches provide effective equipment condition monitoring or personnel position information, they do not directly integrate distance and velocity information for continuous human behavior analysis. For dynamic intrusion scenarios such as approaching and crossing, relying on a single type of spatial or acoustic feature may therefore provide insufficient motion information, particularly in complex environments containing coal piles, equipment structures, and falling objects.

In general acoustic sensing scenarios, acoustic signals have been widely used for indoor localization, gesture recognition, human identification, and fall detection. Gabbrielli et al. [29] developed RAILS, a three-dimensional real-time indoor localization system based on ultrasonic angle-of-arrival estimation. Joo et al. [30] developed an ultrasonic-array-based hand gesture recognition system capable of classifying seven hand gestures, achieving recognition accuracies above 97% across different measurement distances. Xu et al. [31] developed AcousticID, which extracts fine-grained gait information from acoustic signals for human identification. Lian et al. [32] developed an inaudible acoustic sensing system for fall detection, achieving a precision and recall of 92.6%.

Multi-feature and multimodal fusion methods improve target representation by integrating complementary information from different features or sensing modalities [33]. Wei et al. [34] developed a dual-modal feature fusion network that integrates visible and thermal-infrared information to improve object detection in complex dense-forest environments. Liu et al. [35] proposed Adaptively Spatial Feature Fusion (ASFF) to fuse multi-scale features through adaptively learned spatial weights, while Zhao et al. [36] proposed a Multi-Level Feature Pyramid Network (MLFPN) to construct multi-level and multi-scale feature representations for object detection. However, these fusion strategies are primarily designed for visual feature representations and cannot be directly applied to acoustic temporal parameters such as distance and velocity. Millimeter-wave radar provides multidimensional target perception capabilities by exploiting information such as range and velocity [8]; however, radar operates on electromagnetic signals and does not directly address the multipath propagation and vibration-noise characteristics of underground acoustic environments.

## 3. Preliminaries

This section introduces the fundamental principle of the FMCW acoustic sensing system, including the linear frequency-modulated signal model, echo delay relationship, and Doppler velocity measurement mechanism. These theoretical foundations provide the basis for subsequent acoustic echo enhancement and distance–velocity feature extraction.

**Figure 2:**
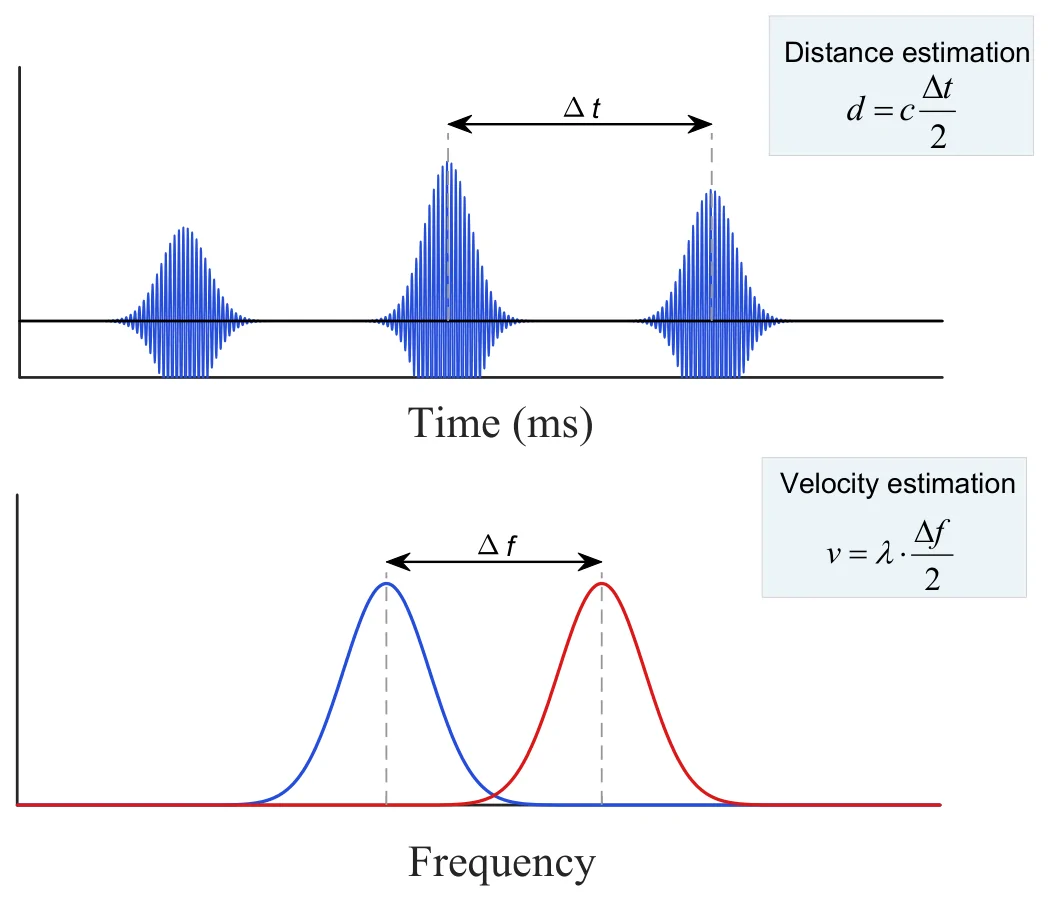
Principle of distance and velocity estimation in the FMCW acoustic sensing system. The target distance is estimated from the time delay Δt between transmitted and received acoustic echoes, while the target velocity is obtained from the Doppler frequency shift Δf.

The fundamental principle of distance and velocity estimation in the FMCW acoustic sensing system is illustrated in Figure 2. The propagation delay Δt between the transmitted signal and the received echo is utilized to estimate the target distance, whereas the Doppler frequency shift Δf is employed to measure the radial velocity of the target.

Frequency-modulated continuous wave (FMCW) acoustic sensing estimates target information by transmitting a linear frequency-modulated Chirp signal and analyzing the frequency difference between the transmitted signal and the received echo. The transmitted acoustic waveform can be expressed as:

(1) $$S_{T}\left(t\right)=A_{T}\cos\left(2\pi f_{c}t+\pi kt^{2}\right)$$

where $A_{T}$ represents the amplitude of the transmitted signal; $f_{c}$ denotes the initial carrier frequency of the Chirp signal; and k=B/T is the frequency modulation slope, where *B* and *T* represent the signal bandwidth and modulation duration, respectively.

The instantaneous frequency of the transmitted Chirp signal is obtained by differentiating the phase term:

(2) $$f\left(t\right)=\frac{1}{2\pi }\frac{d\phi (t)}{dt}=f_{c}+kt$$

The linear variation of instantaneous frequency provides the theoretical basis for target distance estimation through frequency difference measurement.

Owing to the linear frequency modulation, the transmitted Chirp signal is a wideband waveform whose spectral energy is spread over the entire modulation bandwidth *B* rather than being concentrated at a single carrier frequency. By applying the Fourier transform to the analytic form of the transmitted signal, whose real part is given by (1), and invoking the principle of stationary phase (POSP), the spectrum of the Chirp signal can be approximated as:

(3) $${\tilde{S}}_{T}\left(f\right)\approx \frac{A_{T}}{\sqrt{k}}\exp\left[-j\frac{\pi }{k}{\left(f-f_{c}\right)}^{2}+j\frac{\pi }{4}\right],\;f_{c}\leq f\leq f_{c}+B$$

where ${\tilde{S}}_{T}\left(f\right)$ denotes the spectrum of the analytic transmitted signal. The above equation reveals the spectral characteristics of the FMCW signal: within the modulated frequency band $\left[f_{c},f_{c}+B\right]$, the amplitude spectrum is approximately flat with magnitude $A_{T}/\sqrt{k}$ and decays rapidly outside the band, thus forming a nearly rectangular amplitude spectrum of bandwidth *B* (with slight Fresnel ripples near the band edges caused by the finite modulation duration), whereas the phase spectrum varies quadratically with frequency. Such a flat wideband spectrum is a distinctive property of the linear frequency-modulated continuous wave, which uniformly distributes the signal energy over the modulation bandwidth.

Accordingly, the flat wideband spectrum guarantees that, after the dechirp processing described below, the received echo energy is concentrated into a sharp beat-frequency peak, and the achievable range resolution is inversely proportional to the signal bandwidth, i.e., ΔR=c/(2B), which lays the foundation for the high-precision distance estimation of the proposed system.

After propagation and reflection from a target, the received acoustic echo contains a propagation delay caused by the target distance. The received signal can be modeled as:

(4) $$S_{R}\left(t\right)=A_{R}\cos\left(2\pi f_{c}\left(t-\tau \right)+\pi k{\left(t-\tau \right)}^{2}\right)$$

where $A_{R}$ represents the attenuation coefficient caused by propagation loss, and τ denotes the acoustic time-of-flight (ToF) delay, which is defined as:

(5) $$\tau =\frac{2R}{c}$$

where *R* represents the distance between the acoustic sensor and the target, and *c* denotes the acoustic propagation velocity.

By mixing the transmitted signal with the received echo signal and applying a low-pass filter, the high-frequency component can be removed while retaining the intermediate-frequency (IF) component containing distance information:

(6) $$S_{IF}\left(t\right)=A\cos\left(2\pi f_{IF}t+\phi_{c}\right)$$

where $\phi_{c}$ represents the phase shift introduced by propagation delay. The beat frequency is determined by the difference between the transmitted and received instantaneous frequencies:

(7) $$f_{IF}=f_{c}+kt-\left[f_{c}+k\left(t-\tau \right)\right]$$

which can be simplified as:

(8) $$f_{IF}=k\tau =\frac{2kR}{c}$$

Therefore, the target distance can be calculated according to the beat frequency:

(9) $$R=\frac{cf_{IF}}{2k}$$

For moving targets, the received acoustic echo contains an additional frequency shift caused by the Doppler effect. Assuming that the target radial velocity is *v*, the Doppler frequency shift can be expressed as:

(10) $$f_{d}=\frac{2vf_{c}}{c}$$

where $f_{c}$ denotes the carrier frequency of the transmitted Chirp signal. The sign of $f_{d}$ depends on the relative motion direction between the target and the sensor. A positive Doppler shift indicates that the target approaches the sensor, whereas a negative shift indicates that the target moves away.

The beat frequency reflects the spatial position of the target, while the Doppler frequency represents its motion state. Therefore, by extracting the time-varying beat frequency and Doppler components from the received acoustic echo, the target distance and radial velocity can be simultaneously estimated. These complementary distance and velocity features provide the theoretical foundation for the proposed distance–velocity fusion detection framework.

## 4. Architecture Design

### 4.1. Overview

**Figure 3:**
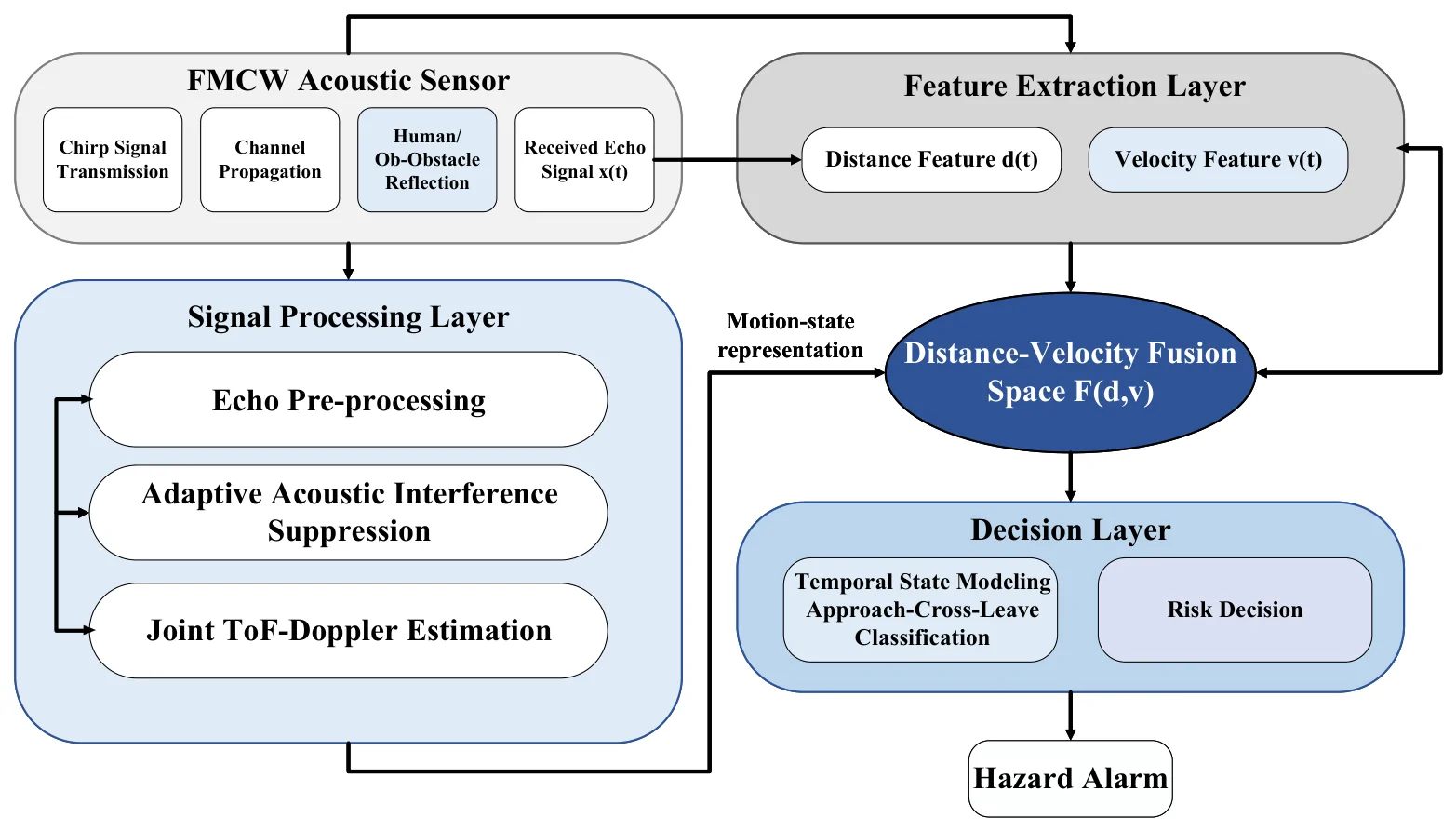
Overall architecture of the proposed ACBSS. The framework consists of three cascaded modules: acoustic interference suppression and echo enhancement module for mitigating multipath interference and attenuation effects, joint distance–velocity feature extraction module for extracting complementary spatial and motion features, and distance–velocity coupled temporal behavior discrimination module for modeling human crossing trajectories and performing risk decisions.

The proposed Acoustic-based Crossing Behavior Sensing System (ACBSS) consists of three cascaded modules, as illustrated in Figure 3.

**Acoustic Interference Suppression and Echo Enhancement Module**: This module aims to mitigate multipath interference and propagation attenuation while suppressing mechanical vibration and background noise, thereby enhancing effective human echo components.

**Joint Distance–Velocity Feature Extraction Module**: Based on acoustic propagation mechanisms, this module jointly estimates the temporal distance variation and radial micro-Doppler velocity of human targets, thereby constructing a complementary distance–velocity feature space.

**Distance–Velocity Coupled Temporal Behavior Discrimination Module**: To overcome the limitation of single-frame detection, a temporal state machine is introduced to characterize the complete “approach–cross–leave” trajectory of human crossing behavior.

### 4.2. Acoustic Interference Suppression and Temporal Alignment

**Figure 4:**
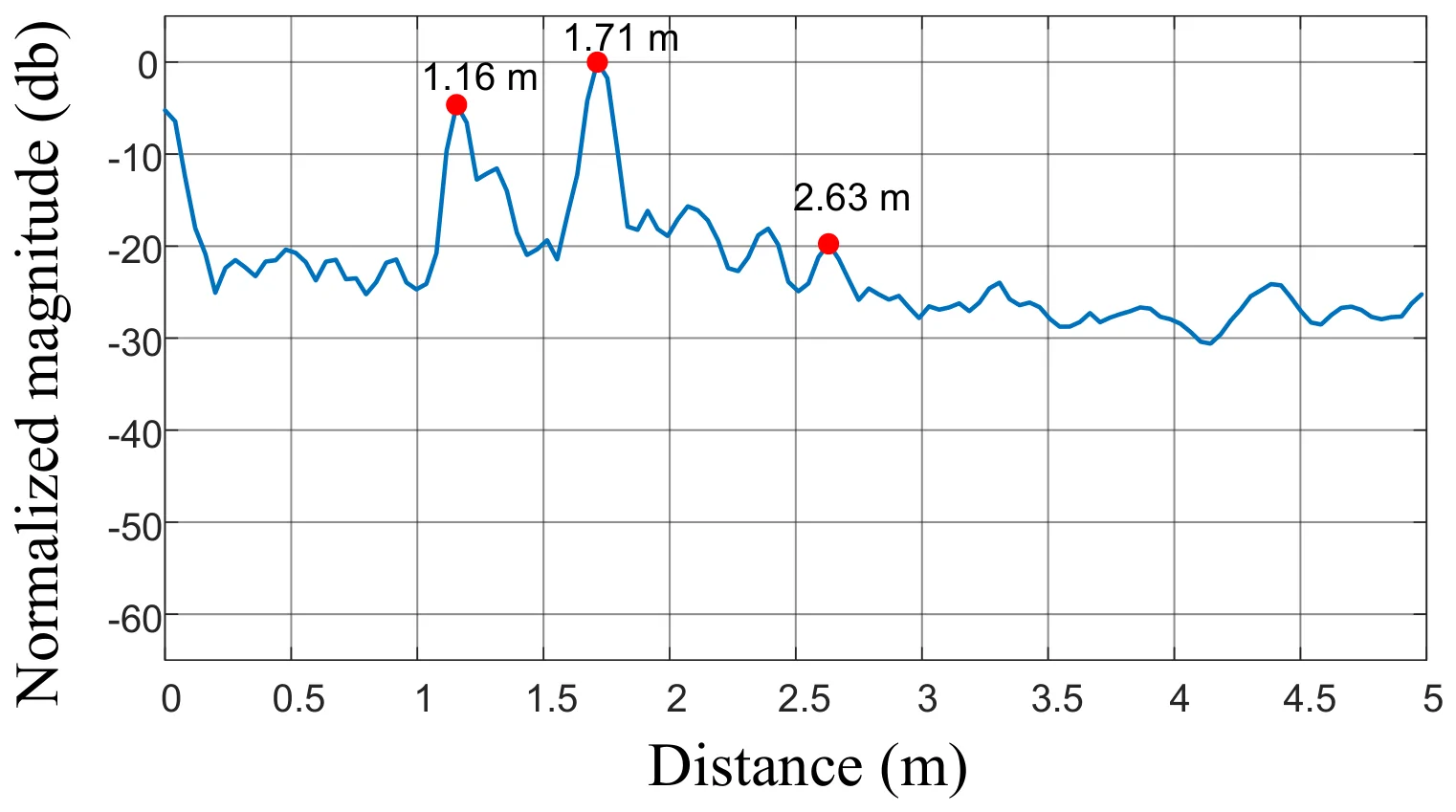
Normalized magnitude distribution of raw acoustic echo signals under underground multipath interference. The peak at 1.71 m represents the real human echo, while peaks at 1.16 m and 2.63 m are multipath ghost echoes induced by tunnel walls, coal piles and conveyor supports.

Underground conveyor tunnels are characterized by strong reverberation, multipath propagation, and mechanical vibration, which significantly reduce the signal-to-noise ratio (SNR) of human echoes and degrade the stability of subsequent distance and velocity estimation. The severe multipath ghost interference phenomenon in raw echo signals is visualized in Figure 4. To improve the robustness of acoustic sensing under such complex environments, a cascaded signal processing strategy consisting of acoustic interference suppression and propagation delay alignment is proposed.

Considering that mechanical vibration and background noise partially overlap with human echoes in the frequency domain, an adaptive spectral interference suppression method based on prior noise information is first employed. The noise power spectral density (PSD) model $P_{noise}\left(f\right)$ is estimated from target-free measurements, and a Wiener filter is applied to suppress stationary interference:

(11) $$H\left(f\right)=\frac{P_{signal}\left(f\right)}{P_{signal}\left(f\right)+\alpha P_{noise}\left(f\right)}$$

where $P_{signal}\left(f\right)$ and $P_{noise}\left(f\right)$ denote the power spectral densities of the received signal and background noise, respectively, and α is an adaptive regularization parameter controlling the trade-off between signal preservation and noise suppression. After Wiener filtering, stationary mechanical interference and broadband background noise are effectively suppressed, producing a cleaner echo signal for subsequent processing.

**Figure 5:**
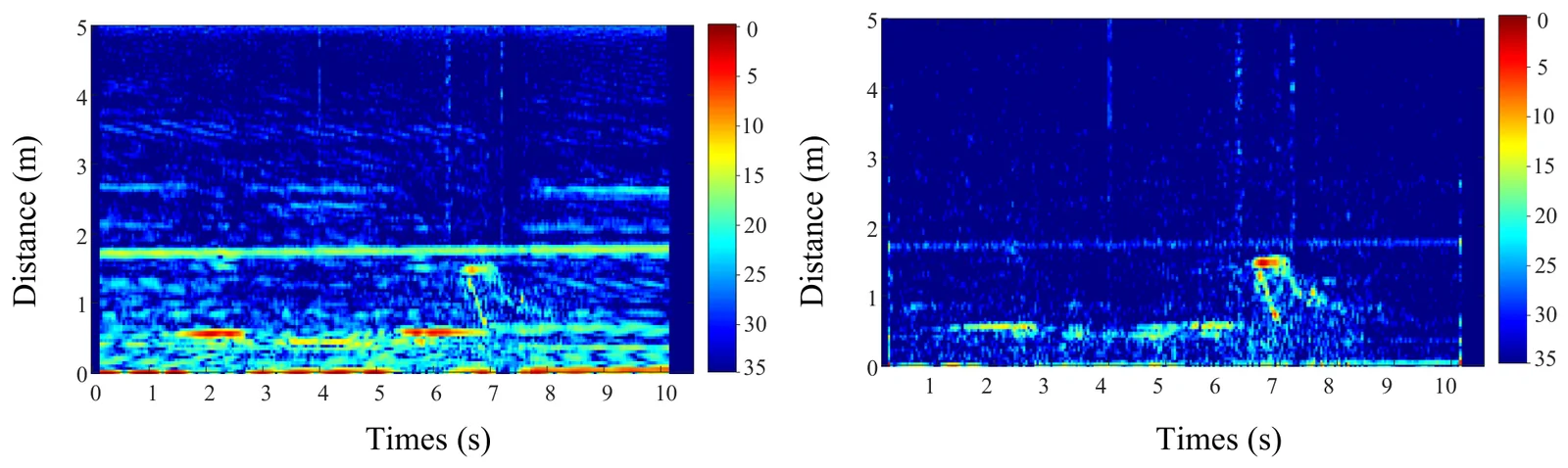
Time-varying distance trajectory of a human crossing the conveyor belt, showing the complete “approach–cross–leave” spatial variation characteristic. Static ghost obstacles only output flat distance curves without continuous change. The (**right**) panel shows the signal-enhanced result of the (**left**) panel after noise removal.

Although spectral interference suppression improves the signal quality, echoes reflected from human targets at different distances still exhibit different propagation delays. Furthermore, multipath propagation introduces delay dispersion, reducing the temporal coherence of the received echo sequence. The continuous time-varying distance trajectory of the human target and the disordered delay distribution of multipath clutter can be observed in Figure 5. To alleviate this problem, a propagation delay alignment strategy is adopted to compensate for the estimated propagation delay of the dominant target echo.

Let $\hat{\tau }\left(t\right)$ denote the propagation delay estimated from the peak of the matched-filter output. The aligned echo signal is obtained by

(12) $$S_{aligned}\left(t\right)=S_{decoupled}\left(t+\hat{\tau }\left(t\right)\right)$$

where $\hat{\tau }\left(t\right)$ is the estimated propagation delay corresponding to the dominant target echo. This operation aligns echoes originating from different target distances to a common temporal reference without modifying the physical propagation path.

After temporal alignment, the delay dispersion caused by multipath propagation is effectively reduced, leading to improved temporal coherence of the target echo sequence. As a result, both the ToF-based distance estimation and Doppler feature extraction become more stable under complex underground acoustic environments, providing reliable input for the subsequent spatiotemporal dual-feature estimation and crossing behavior recognition.

### 4.3. Spatiotemporal Orthogonal Dual-Feature Joint Estimation

The crossing behavior of personnel near conveyor belts is essentially a physical process involving both spatial occupation and continuous motion. To comprehensively characterize this behavior, the proposed method extracts two complementary features from the perspectives of spatial localization and motion representation, namely the temporal distance trajectory and radial velocity variation.

The spatial feature is estimated based on the time-of-flight (ToF) principle. Assuming a constant acoustic propagation velocity during each sensing period, the target distance is calculated as

(13) $$d\left(t\right)=\frac{c\tau_{peak}\left(t\right)}{2}$$

where *c* denotes the acoustic propagation velocity and $\tau_{peak}\left(t\right)$ represents the propagation delay corresponding to the dominant echo peak extracted after signal enhancement. The obtained distance trajectory is denoted as D(t), which characterizes the temporal variation of the target position.

During conveyor belt crossing, the movement of the human torso and limbs introduces micro-Doppler modulation into the acoustic echo. By applying short-time Fourier transform (STFT) to the enhanced echo signal, the time-frequency representation matrix is obtained as

(14) $$S\left(f,t\right)=STFT\{S_{enhanced}\left(t\right)\}$$

The instantaneous Doppler frequency shift $f_{d}\left(t\right)$ is extracted from the energy ridge of the time-frequency distribution. According to the Doppler principle introduced in Section 3, the radial velocity of the target is estimated by

(15) $$v\left(t\right)=\frac{cf_{d}\left(t\right)}{2f_{c}\cos\left(\theta \right)}$$

where $f_{c}$ denotes the center frequency of the transmitted Chirp signal and θ represents the acoustic incident angle.

To suppress velocity fluctuations caused by conveyor vibration and residual noise, a Kalman filter is employed to smooth the estimated velocity sequence:

(16) V(t)=KF{v(t)}

where V(t) denotes the filtered radial velocity sequence.

**Figure 6:**
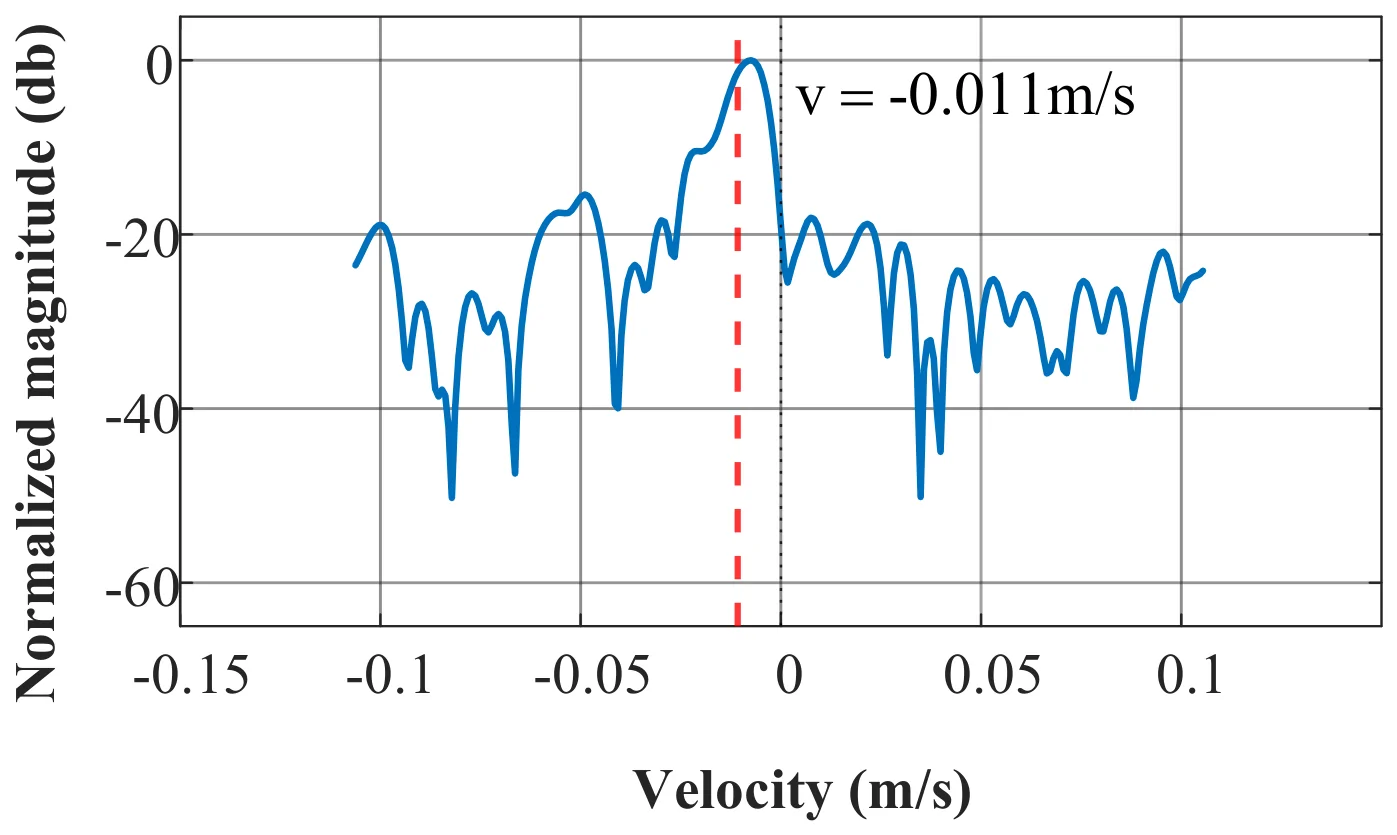
Normalized magnitude spectrum of Doppler velocity for mechanical vibration interference. The tiny radial velocity v=−0.011 m/s corresponds to low-amplitude transient clutter rather than human limb micro-Doppler motion. The blue solid line represents the normalized magnitude spectrum, while the red dashed line indicates the estimated target velocity (v=−0.011 m/s).

By combining ToF-based distance estimation with micro-Doppler velocity estimation, the proposed framework simultaneously obtains the spatial distance feature D(t) and motion feature V(t). As illustrated in Figure 6, mechanical vibration can introduce spurious low-magnitude velocity components in the estimated motion feature V(t). These complementary features provide the basis for the subsequent distance–velocity coupled behavior discrimination.

### 4.4. Distance–Velocity Coupled Temporal Behavior Discrimination

**Figure 7:**
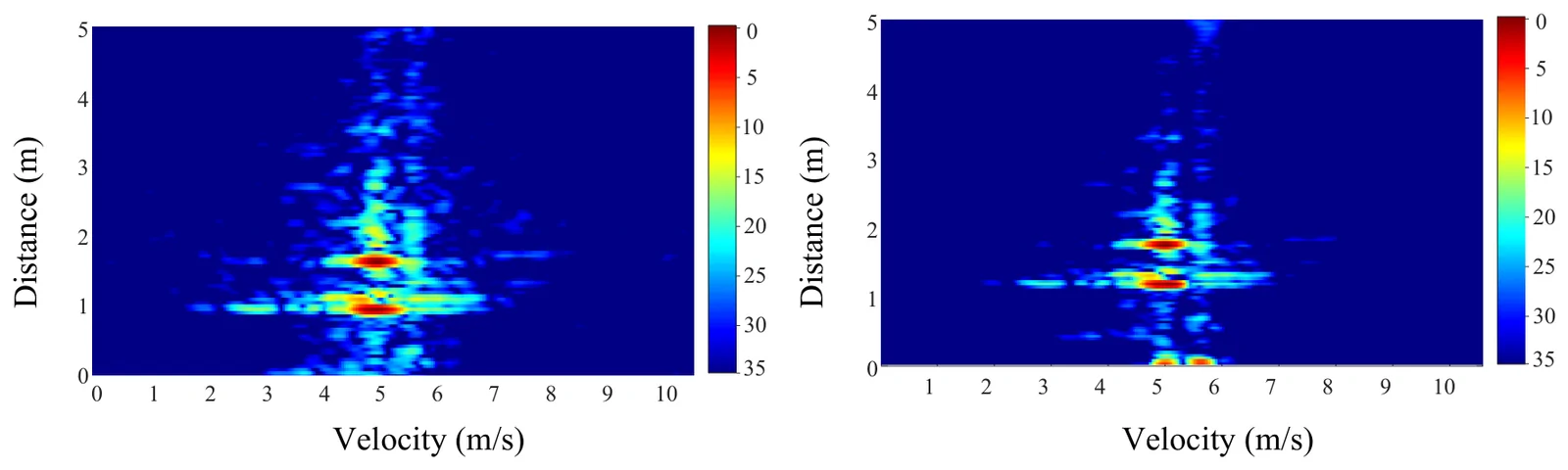
2D distance–velocity feature distribution space for distinguishing human targets, static ghost echoes and mechanical vibration clutter. Dynamic human crossing presents continuous coupled variation of distance and radial velocity. The (**right**) panel shows the signal-enhanced result of the (**left**) panel after noise removal.

Relying solely on distance or velocity is insufficient for accurate crossing detection; static obstacles yield stable distance responses without velocity variations, while conveyor vibrations cause transient Doppler shifts without meaningful spatial displacement. These different signal manifestations among targets and interferences can be observed in Figure 7. To address this, we propose a distance–velocity coupled temporal discrimination strategy. First, the extracted distance trajectory D(t) and radial velocity sequence V(t) are normalized to eliminate dimensional differences:

(17) $$x_{d}\left(t\right)=\frac{d\left(t\right)-\mu_{d}}{\sigma_{d}},\;x_{v}\left(t\right)=\frac{v\left(t\right)-\mu_{v}}{\sigma_{v}}$$

where μ and σ denote the mean and standard deviation within a sliding window. Given that feature reliability varies with environmental interference, an adaptive confidence weighting strategy is adopted. The confidence coefficients for distance and velocity are defined inline as $C_{d}\left(t\right)=SNR_{d}\left(t\right)/\left[SNR_{d}\left(t\right)+SNR_{v}\left(t\right)\right]$ and $C_{v}\left(t\right)=SNR_{v}\left(t\right)/\left[SNR_{d}\left(t\right)+SNR_{v}\left(t\right)\right]$, where SNR represents the feature quality indicator. The fused confidence score is then calculated as:

(18) $$S\left(t\right)=C_{d}\left(t\right)f_{d}\left(x_{d}\left(t\right)\right)+C_{v}\left(t\right)f_{v}\left(x_{v}\left(t\right)\right)$$

where $f_{d}\left(\cdot \right)$ and $f_{v}\left(\cdot \right)$ are feature activation functions mapping the normalized features into the confidence space.

**Figure 8:**
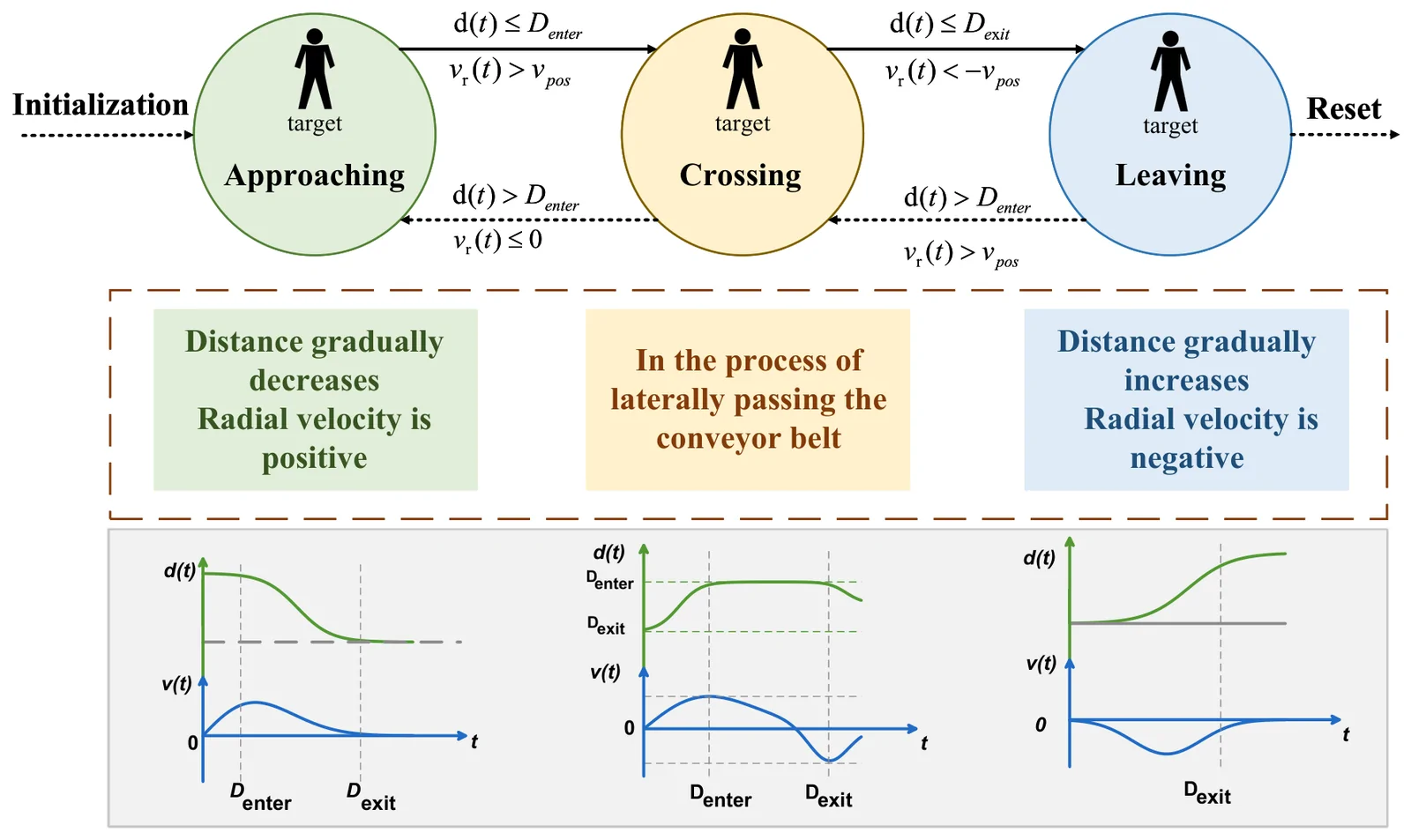
Temporal state machine for human crossing behavior recognition. The target motion is modeled as three sequential states, including approaching, crossing, and leaving. The state transitions are determined by the distance variation d(t) and radial velocity $v_{r}\left(t\right)$, enabling continuous trajectory modeling beyond single-frame hazard detection. The green and blue curves represent the target distance d(t) and relative velocity v(t), respectively.

Human crossing is a continuous spatiotemporal process. To exploit this temporal continuity, a three-state temporal state machine (TSM) is established: $S=\{S_{app},S_{cro},S_{lea}\}$, representing the approaching, crossing, and leaving states, respectively. The overall state transition process is illustrated in Figure 8. The state transitions are governed by specific kinematic rules.

In $S_{app}$, the target approaches the sensing region, where the distance continuously decreases and the radial velocity satisfies v(t)>0.

In $S_{cro}$, the target passes through the sensing area laterally, where the distance reaches its minimum and the velocity gradually changes sign.

In $S_{lea}$, the target moves away from the sensing region, where the distance gradually increases and the radial velocity satisfies v(t)<0.

A crossing event is confirmed only when the trajectory satisfies the complete state transition:

(19) $$\Phi_{valid}=S_{app}\to S_{cro}\to S_{lea}$$

within a predefined time window ΔT. Additionally, the trajectory must satisfy the following spatial and motion constraints:

(20) $$d_{min}<d_{belt},\;\left|v\left(t\right)\right|>v_{th}$$

where $d_{belt}$ is the hazardous boundary of the conveyor belt and $v_{th}$ is the minimum valid human velocity threshold. This joint spatiotemporal constraint effectively suppresses false alarms from static obstacles and transient interference.

Finally, to adapt to non-stationary underground noise, an adaptive threshold updating mechanism is introduced:

(21) $$\theta_{enter}\left(t\right)=\mu_{noise}\left(t\right)+k\sigma_{noise}\left(t\right)$$

where $\mu_{noise}$ and $\sigma_{noise}$ are the mean and standard deviation of the background fusion score estimated within a noise observation window, and *k* is a safety coefficient. To prevent accidental false alarms, a continuous-frame confirmation strategy is employed. The final alarm decision is expressed as:

(22) $$Alarm\left(t\right)=\left\{\begin{array}{cc} 1, & \sum_{i=t-N_{c}+1}^{t}I\left(S_{cro}\left(i\right)\right)\geq N_{c}\;\mathrm{and}\;S\left(t\right)>\theta_{confirm}\\ 0, & \mathrm{otherwise} \end{array}\right.$$

where I(·) is the indicator function and $N_{c}$ is the confirmation window length. By integrating adaptive fusion, TSM transitions, and dynamic thresholding, the framework robustly distinguishes true crossings from environmental noise and mechanical vibrations.

### 4.5. Parameter Determination and Implementation Details

To improve the reproducibility of the proposed method, the main algorithmic parameters and their determination criteria are summarized in Table 1. The parameters were determined according to either physical constraints or calibration measurements. Specifically, the hazardous distance $d_{\mathrm{belt}}$ was defined according to the geometry of the monitored conveyor region, while the velocity threshold $v_{\mathrm{th}}$ was selected above the dominant velocity fluctuations caused by conveyor vibration. The STFT and Kalman filter parameters were selected to provide stable Doppler estimation while preserving the temporal variation of human motion.

The remaining decision parameters were determined from calibration recordings containing target-free background signals, non-crossing interference, and complete “approach–cross–leave” trajectories. The coefficient *k* controls the adaptive background threshold in Equation (21), whereas ΔT and $N_{c}$ were selected according to the duration of valid crossing trajectories and transient disturbances, respectively. After calibration, all parameters were fixed and remained unchanged for experiments under different sensing distances, motion directions, noise levels, and conveyor loading conditions. No parameter was individually retuned on the test samples.

## 5. Performance Evaluation

To evaluate the effectiveness and robustness of the proposed acoustic echo-based distance–velocity fusion method, extensive experiments were conducted on a laboratory conveyor platform under simulated underground environmental conditions, focusing on detection performance under varying sensing distances, motion directions, noise levels, and conveyor operating conditions.

### 5.1. Experimental Setup and Evaluation Metrics

**Figure 9:**
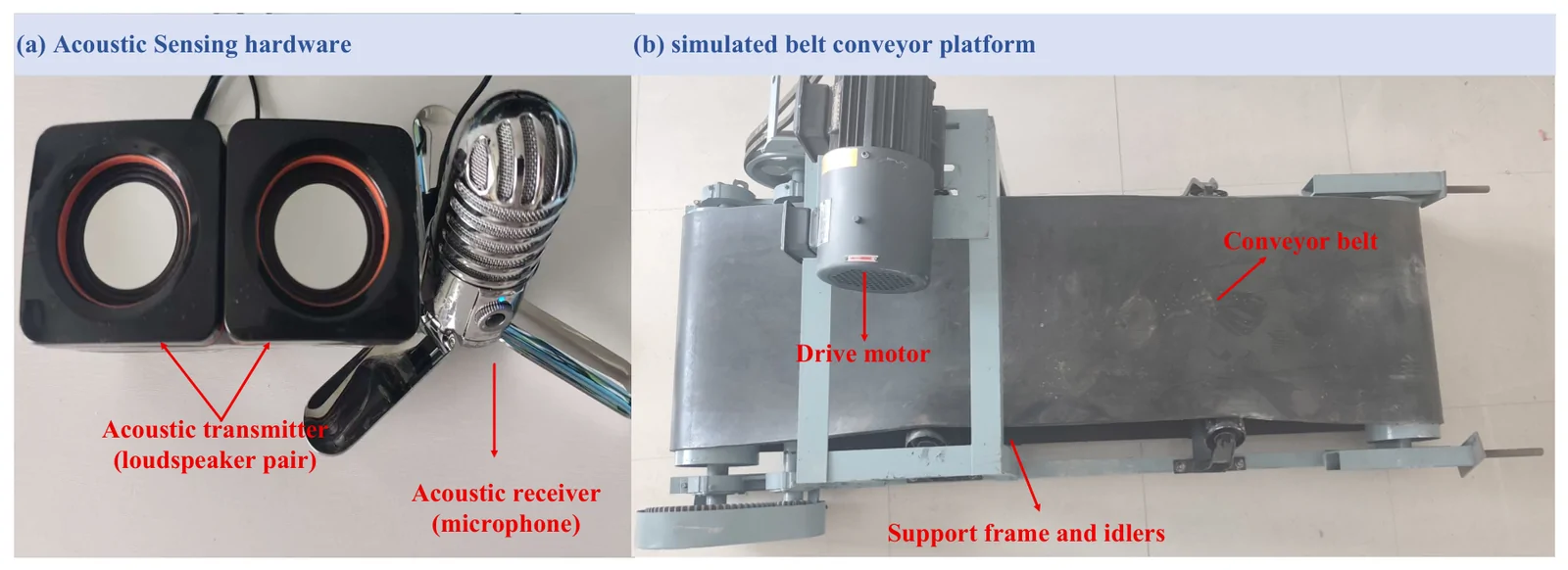
Experimental setup of the simulated underground conveyor belt platform: (**a**) acoustic sensing hardware, including a pair of loudspeakers as the acoustic transmitter and a microphone as the acoustic receiver; (**b**) simulated belt conveyor platform, including the drive motor, conveyor belt, support frame, and idlers. The platform reproduces typical underground monitoring conditions, including confined space, acoustic reflection, mechanical vibration, and environmental interference.

To evaluate the effectiveness of the proposed acoustic echo-based distance–velocity fusion method, a simulated underground conveyor belt platform was constructed, as shown in Figure 9. The platform was designed to reproduce typical underground monitoring conditions, including confined space, acoustic reflection, mechanical vibration, and background interference.

Experiments were conducted on a 2-m-long conveyor belt platform. The collected data were manually annotated according to synchronized video recordings. The experimental sensing system consisted of one acoustic transmitting unit and one receiving unit mounted on the same side of the conveyor monitoring area. The transmitting unit comprised a pair of commercial loudspeakers, while an omnidirectional condenser microphone was used as the receiver. A quasi-monostatic configuration was adopted, with a TX–RX baseline of approximately 10 cm, and the acoustic transceiver was oriented toward the personnel crossing region to ensure that the main propagation path covered the hazardous area beside the conveyor belt. During data acquisition, FMCW chirps covering 18–22 kHz were continuously transmitted with a sampling frequency of 48 kHz and a chirp duration of 40 ms. The received echoes were synchronously recorded and manually annotated using video recordings. The raw acoustic signals were subsequently processed through echo preprocessing, adaptive acoustic interference suppression, ToF–Doppler joint estimation, STFT-based velocity extraction, and Kalman filtering, followed by distance–velocity feature fusion and temporal-state recognition.

The key hardware specifications and signal processing parameters of the proposed FMCW acoustic sensing system are summarized in Table 2. The system operates in the near-ultrasonic band (18–22 kHz) to avoid human auditory discomfort while providing a 4 kHz bandwidth for sufficient range resolution. A quasi-monostatic geometric arrangement with a 10 cm baseline between the commercial loudspeaker pair and the condenser microphone is adopted to balance transceiver isolation and echo reception efficiency. The detailed configurations of the acoustic field and the temporal alignment parameters are strictly aligned with the theoretical derivations in Section 3.

To further examine the dependence of the detection performance on the main empirical parameters, a sensitivity analysis was performed around the nominal parameter settings. The analysis focuses on $d_{\mathrm{belt}}$, $v_{\mathrm{th}}$, the temporal window ΔT, and the adaptive fusion mechanism, which directly affect spatial constraint, motion validation, temporal-state transition, and feature fusion, respectively. As summarized in Table 3, moderate variations around the nominal settings do not change the basic crossing-decision mechanism. In contrast, excessively small or large values may increase false alarms or missed detections. In particular, $d_{\mathrm{belt}}$ mainly controls the spatial extent of the hazardous region, $v_{\mathrm{th}}$ determines the rejection of low-velocity mechanical disturbances, and ΔT controls the tolerance to different crossing durations. For adaptive fusion, the SNR-based weights vary continuously with feature quality rather than relying on a fixed empirical weighting coefficient. Therefore, the proposed method is not determined by a single narrowly tuned fusion weight, while the physically meaningful thresholds should remain within reasonable ranges consistent with the monitored conveyor geometry and human motion characteristics.

To ensure the reliability of the evaluation and address the complexity of real underground environments, the simulated platform was specifically configured to replicate key physical parameters of actual coal mines, including acoustic noise spectra, mechanical vibrations, and ambient conditions. The specific configurations are detailed as follows:

Instead of using simple white noise, the background interference was simulated using audio recorded from an actual underground coal mine (synthesized based on real mine noise profiles). The spectral composition specifically includes low-frequency mechanical hums from motors and idlers (typically <500 Hz) and high-frequency transient impacts from coal collisions (ranging from 1 kHz to 4 kHz). As illustrated in the noise spectrum analysis, the simulated acoustic interference effectively covers the dominant frequency bands encountered in real underground environments.

The mechanical vibration of the 2 m conveyor platform was measured using an accelerometer (Model: PCB 352C33). Under medium- and full-load conditions, the root mean square (RMS) vibration velocity of the conveyor frame was maintained at approximately 2.5 to 4.2 mm/s. This vibration level is consistent with the mechanical vibration characteristics of typical underground belt conveyors, ensuring the robustness of the proposed algorithm against structural interference.

Due to safety regulations and facility constraints in the laboratory, physical coal dust (measured in mg/m^3^) and strong ventilation airflow could not be directly replicated in the current setup. The ambient temperature and humidity were controlled at 22 ± 2 °C and 55 ± 5% RH, respectively. Although acoustic sensing is independent of illumination and is generally less susceptible to airborne dust than optical and infrared sensing, suspended particles may still cause acoustic attenuation and scattering, particularly in the near-ultrasonic frequency range used in this study. Therefore, the robustness of the proposed system under high-concentration coal dust has not been experimentally validated and requires further investigation under real underground conditions. For the current laboratory experiments, a constant acoustic velocity of 340 m/s was adopted for ToF calculations. The potential influence of environmental variations on sound velocity and ranging accuracy is further discussed in Section 6.4. The present experiments primarily validate the robustness of the proposed method against complex acoustic noise and mechanical vibrations under controlled laboratory conditions. Comprehensive on-site tests incorporating realistic dust concentrations, ventilation airflow, and environmental variations will be conducted in future work.

The detection task is formulated as a binary classification problem, where personnel crossing events are regarded as positive samples and other behaviors are treated as negative samples. Accuracy, Precision, Recall, and F1-score are adopted as evaluation metrics.

Accuracy is calculated as:

(23) $$Accuracy=\frac{TP+TN}{TP+TN+FP+FN}\times 100\%$$

Precision is defined as:

(24) $$Precision=\frac{TP}{TP+FP}\times 100\%$$

Recall is calculated as:

(25) $$Recall=\frac{TP}{TP+FN}\times 100\%$$

The F1-score is calculated as:

(26) $$F1\text{-}score=\frac{2\times Precision\times Recall}{Precision+Recall}\times 100\%$$

where TP, TN, FP, and FN denote true positive, true negative, false positive, and false negative cases, respectively.

### 5.2. Ablation Study of Distance–Velocity Fusion

To verify the effectiveness of the proposed distance–velocity fusion strategy, an ablation study was conducted under the standard crossing scenario with a sensing distance of 2 m. Three different feature configurations were compared: **Distance-only**, which performs recognition based only on the ToF distance feature; **Velocity-only**, which relies exclusively on the Doppler velocity feature; and the **Proposed** method, which jointly utilizes distance and velocity information with temporal constraints.

The comparison results are summarized in Table 4. The proposed method achieved an accuracy of 96.5%, a precision of 96.8%, a recall of 95.6%, and an F1-score of 96.2%. In comparison, the Distance-only method obtained a relatively high recall of 93.6%, but its precision was limited to 78.4%. This indicates that relying solely on spatial distance information may lead to false alarms when non-crossing targets exhibit distance characteristics similar to actual crossing events. The Velocity-only method achieved a precision of 91.5%, but its recall decreased to 81.2%, indicating that velocity information alone may miss crossing events when motion features are affected by conveyor vibration or environmental disturbances.

**Figure 10:**
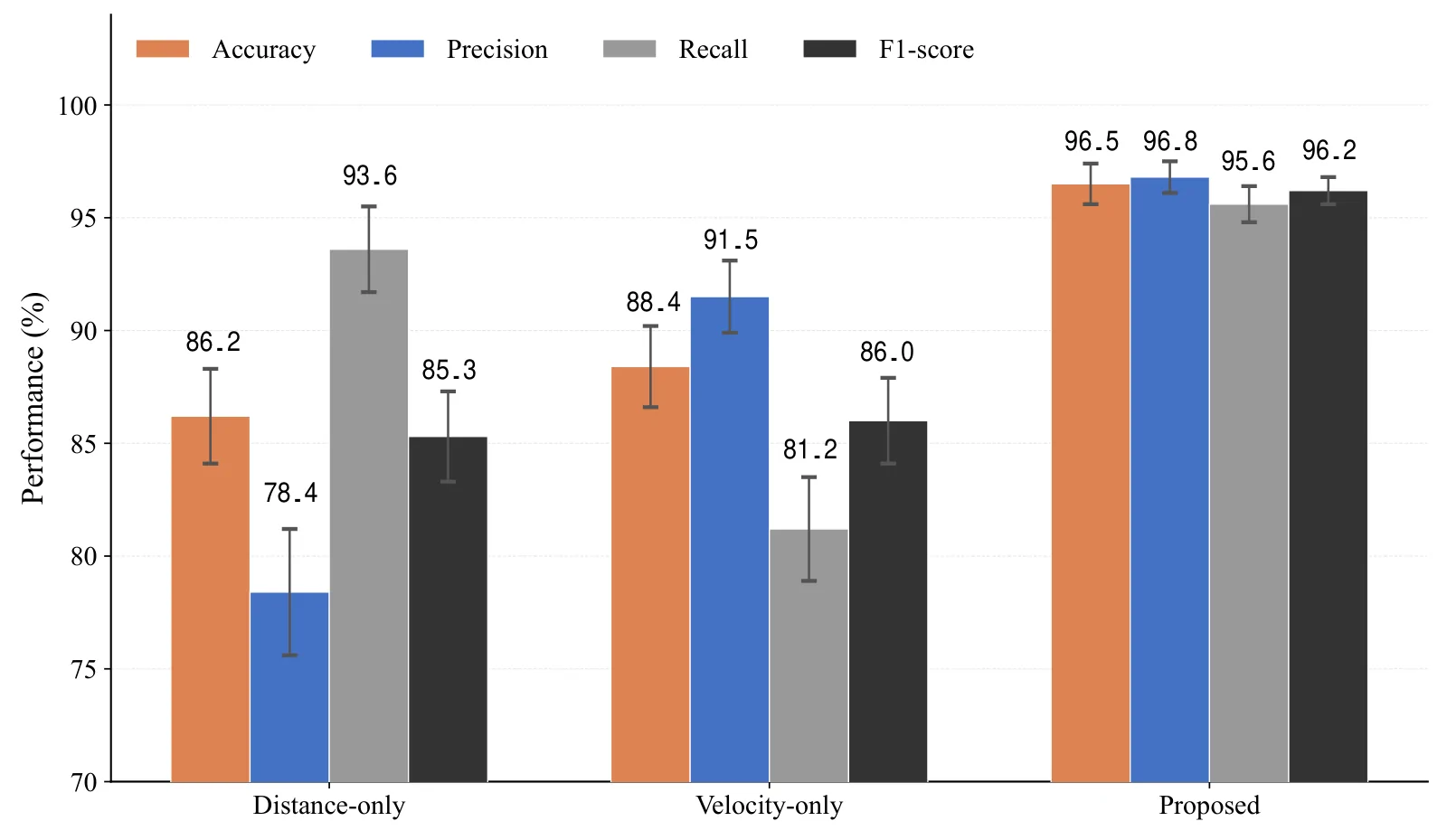
Performance comparison of different feature combinations under the standard crossing scenario. The proposed distance–velocity fusion method achieves higher overall performance than the Distance-only and Velocity-only configurations. Error bars indicate the standard deviation across repeated tests.

As illustrated in Figure 10, the proposed method consistently achieves higher Accuracy, Precision, Recall, and F1-score than the two single-feature configurations. These results demonstrate the complementary roles of distance and velocity information: the distance feature characterizes the spatial relationship between the target and the conveyor belt, whereas the velocity feature reflects dynamic motion characteristics. Their joint utilization reduces the ambiguity associated with individual features and improves the overall robustness of crossing detection.

### 5.3. Influence of Sensing Distance and Motion Direction

To evaluate the spatial adaptability of the proposed method, experiments were conducted under different sensing distances and target motion directions. The crossing distance was varied from 1 m to 4 m, while different relative motion directions were tested at a fixed distance of 2 m.

**Figure 11:**
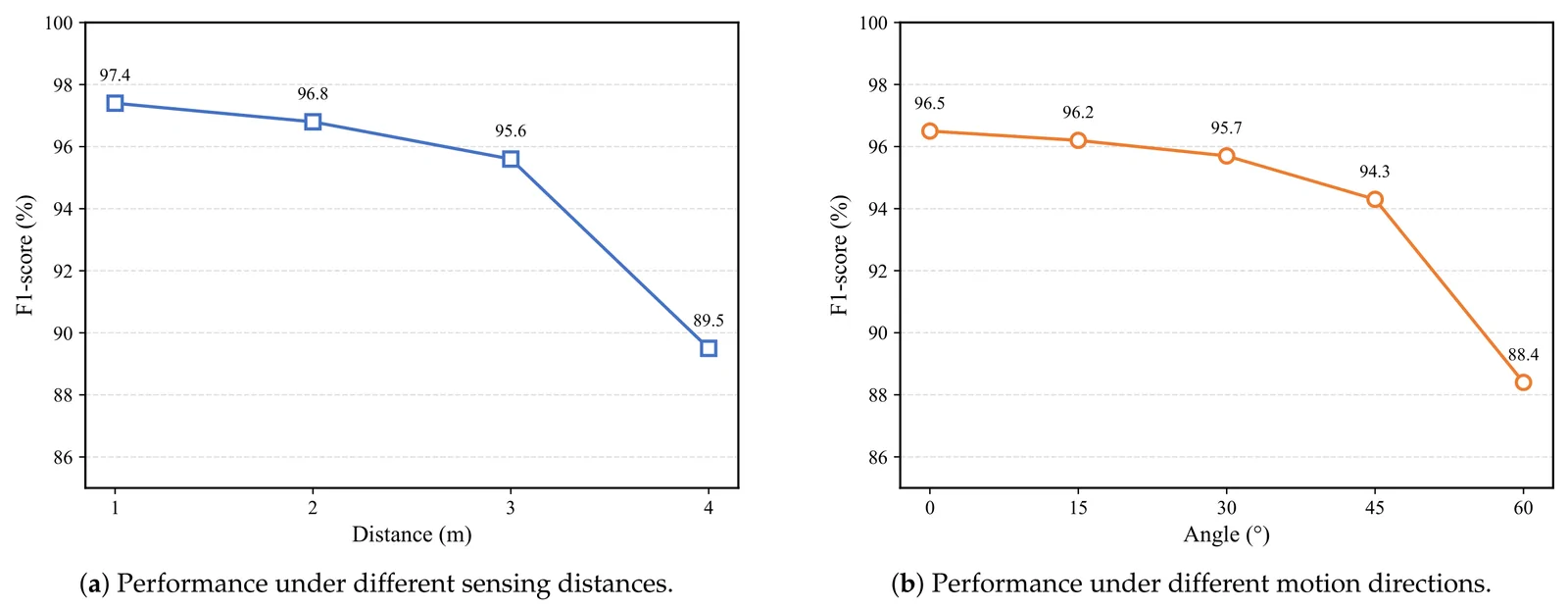
Performance analysis under different sensing distances and motion directions.

As shown in Figure 11a, the proposed method maintains stable performance within 1–3 m. When the distance increases to 4 m, the performance decreases slightly due to acoustic attenuation and reduced echo SNR. However, the method still satisfies the requirements of conveyor belt safety monitoring.

Figure 11b presents the influence of motion direction. The performance remains stable under moderate directional variations, while larger changes reduce the radial velocity component and weaken Doppler features, resulting in slight degradation.

**Figure 12:**
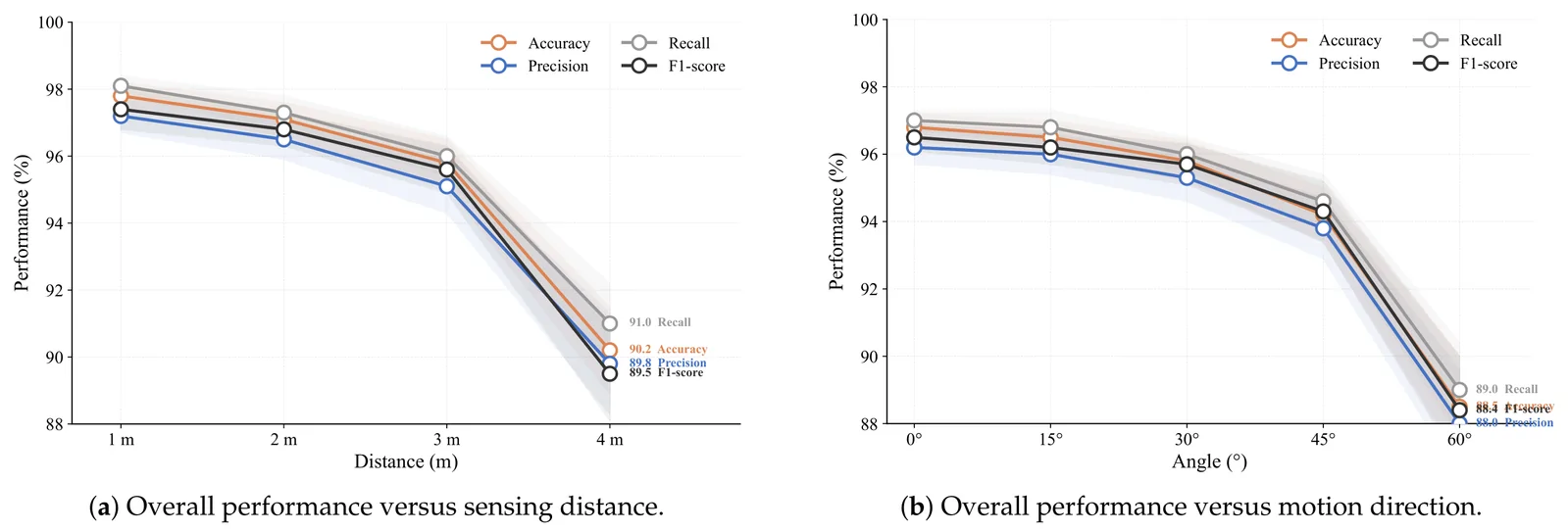
Overall detection performance under different sensing distances and motion directions: (**a**) performance versus sensing distance; (**b**) performance versus motion direction. Accuracy, precision, recall, and F1-score are reported for both conditions.

The overall performance under different distances and motion directions is summarized in Figure 12. The proposed method achieves an F1-score above 95% within the main monitoring range and maintains reliable detection performance under different target positions and motion conditions.

### 5.4. Impact of Ambient Noise and Belt Load

The acoustic characteristics of conveyor operation vary significantly under different environmental conditions and loading states. Therefore, experiments were conducted under different ambient noise levels as well as empty-, medium-, and full-load conditions at a sensing distance of 2 m.

As summarized in Table 5, the proposed method achieved F1-scores above 95% under empty-load (approximately 65 dB) and medium-load (approximately 75 dB) conditions. Under full-load conditions, where mechanical noise increased to approximately 85 dB and transient impact noises occurred due to coal collision, the F1-score decreased slightly to 91.4%.

**Figure 13:**
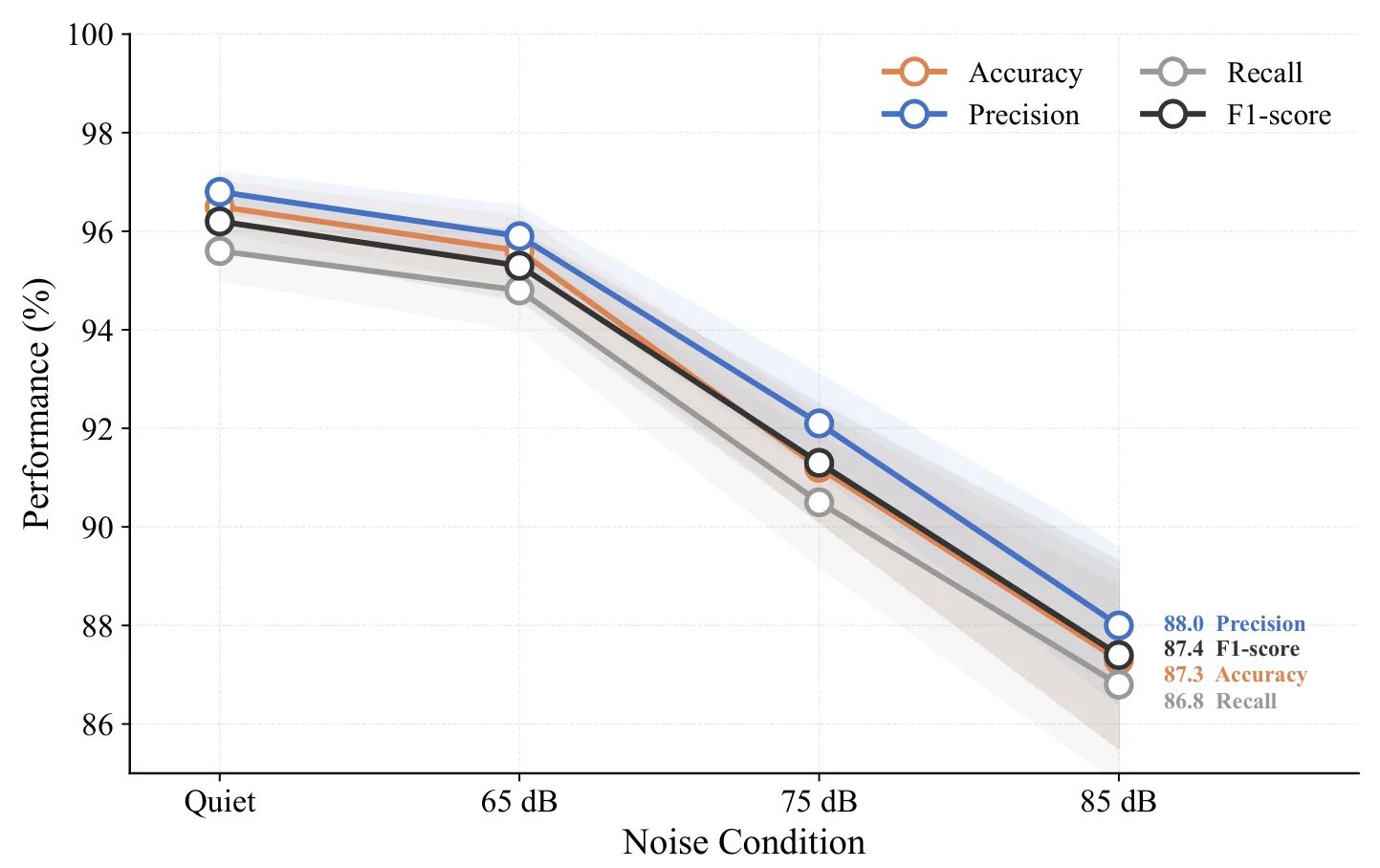
Detection performance under different ambient noise levels.

As illustrated in Figure 13, the proposed method maintained stable detection performance under low- and medium-noise environments. Although the F1-score decreased under severe acoustic interference, the adaptive confidence weighting strategy effectively suppressed transient disturbances and preserved robust behavior recognition.

**Figure 14:**
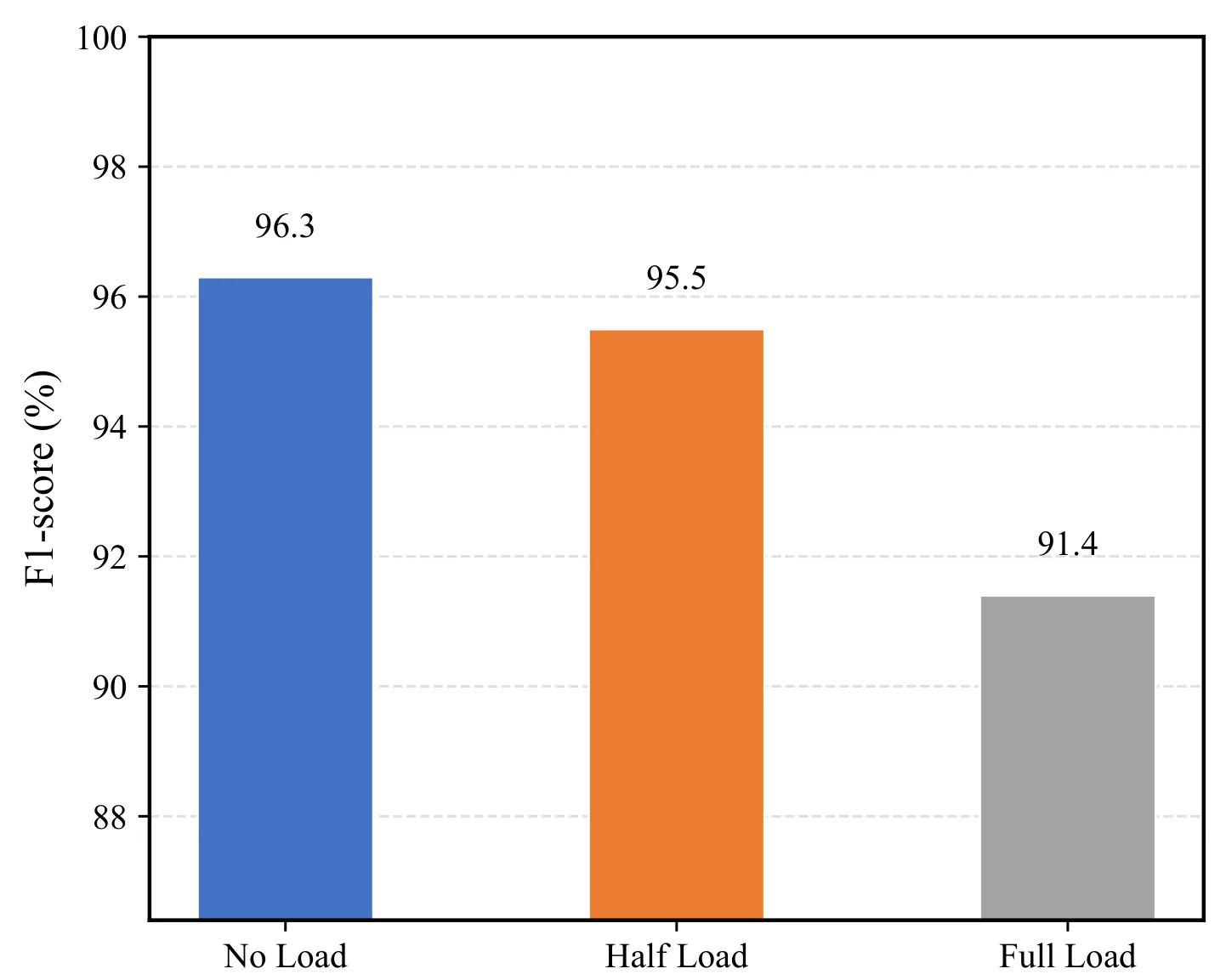
Detection performance under different conveyor belt loading conditions.

To further investigate the influence of conveyor operating conditions, experiments were conducted under different belt loading states. The corresponding results are presented in Figure 14. As the conveyor load increased from empty-load to full-load, stronger structural vibration and impact noise gradually reduced the echo SNR, leading to a slight degradation in detection performance. Nevertheless, the proposed method consistently achieved reliable recognition accuracy owing to the continuous-frame confirmation mechanism.

Overall, these results demonstrate that the proposed distance–velocity fusion framework maintains high robustness under non-stationary underground acoustic environments, making it suitable for practical conveyor belt safety monitoring.

### 5.5. Experiments in a Simulated Underground Tunnel Environment

To further evaluate the practical applicability of the proposed method, experiments were conducted in a simulated coal mine roadway environment. The platform reproduced typical underground characteristics, including confined space, acoustic reflections, limited illumination, and environmental interference. The acoustic sensing device was deployed near the conveyor belt to collect target and background echo signals.

A total of 500 samples were collected to evaluate the detection performance under different experimental conditions. The proposed method was compared with infrared beam and low-light vision detection methods. As shown in Table 6, low-light vision achieved a recall of 76.5% due to image degradation under weak illumination and environmental interference. Infrared beam detection obtained a precision of 68.2%, reflecting its limited ability to distinguish parallel walking behaviors from actual crossing events. In comparison, the proposed distance–velocity fusion method achieved an accuracy of 93.8% and an F1-score of 93.5%.

For the 500 experimental samples, the target trajectories were designed to cover representative personnel motions around the conveyor belt rather than a single fixed crossing path. The crossing samples consisted of complete “approach–cross–leave” trajectories, in which the participant approached the monitored region, crossed the conveyor hazard boundary, and subsequently moved away from the sensing area. To evaluate the influence of target orientation, relative motion directions of 0°, 15°, 30°, 45°, and 60° were considered, with directional tests conducted at a representative sensing distance of 2 m. The sensing distance was additionally varied from 1 to 4 m to cover different target positions. Participants moved at natural walking speeds rather than at a prescribed constant velocity; therefore, the instantaneous radial velocity varied continuously during each trajectory and was estimated directly from the Doppler shift. The 300 non-crossing interference samples included target-free background conditions and representative disturbances without a complete crossing trajectory, allowing the evaluation to distinguish actual conveyor crossing from approaching behavior, static interference, and environmental disturbances.

**Figure 15:**
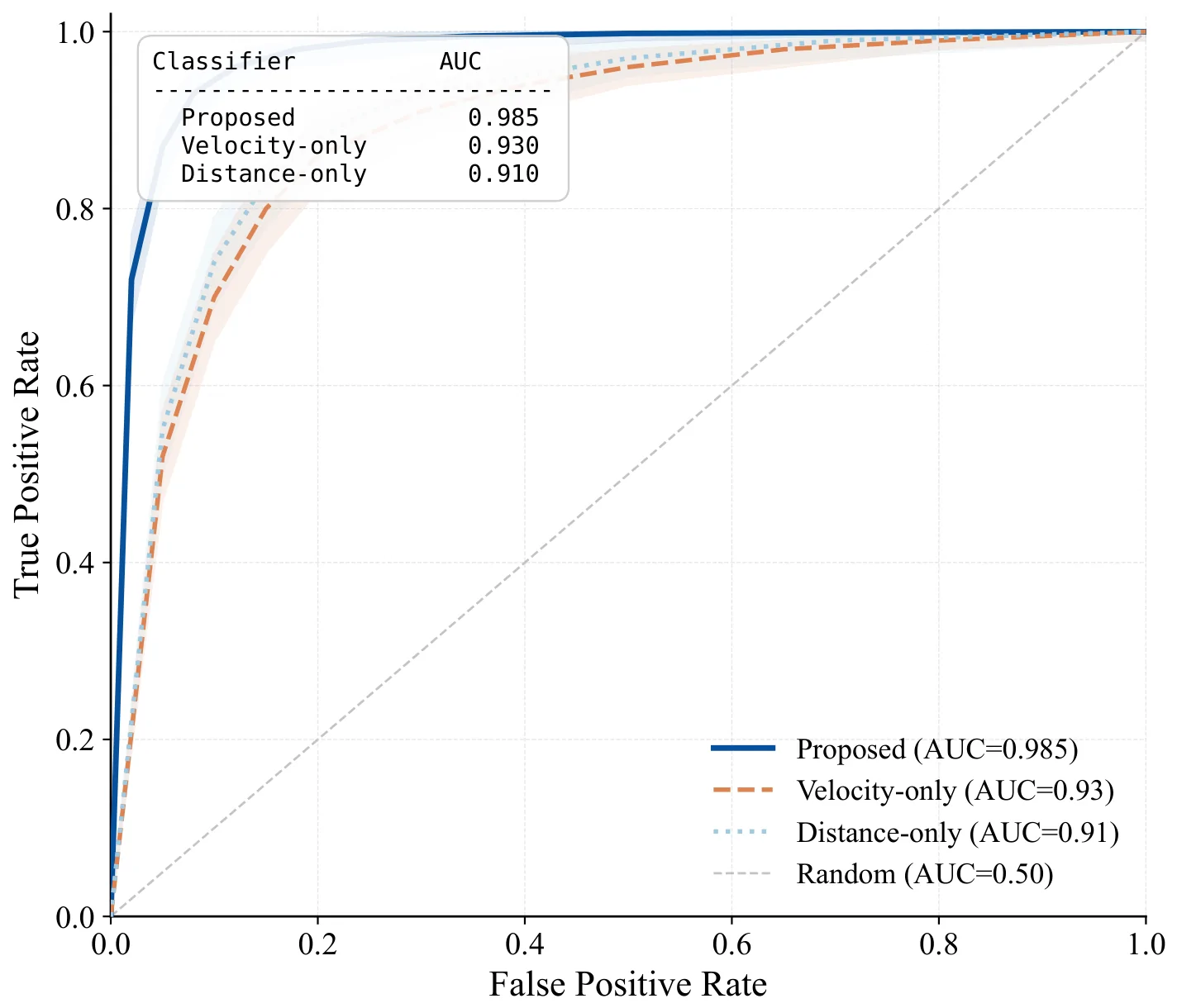
ROC curves of different personnel detection methods in the simulated underground tunnel environment.

**Figure 16:**
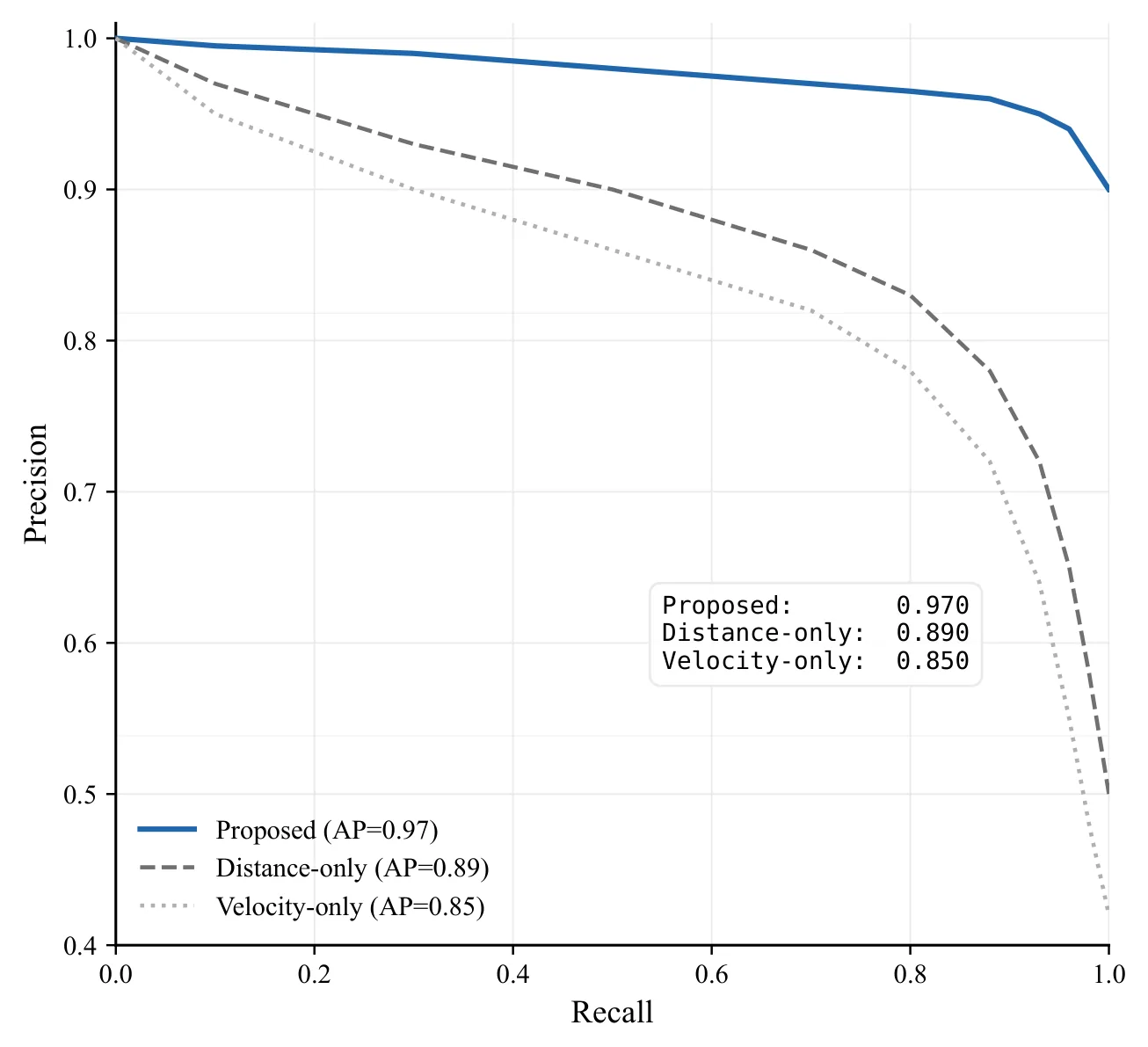
Precision–Recall curves of different personnel detection methods.

The Receiver Operating Characteristic (ROC) and Precision–Recall (PR) curves are presented in Figure 15 and Figure 16. The proposed method achieves higher AUC and average precision than the Distance-only and Velocity-only methods, demonstrating the improved discrimination capability and robustness of the proposed distance–velocity feature fusion strategy.

**Figure 17:**
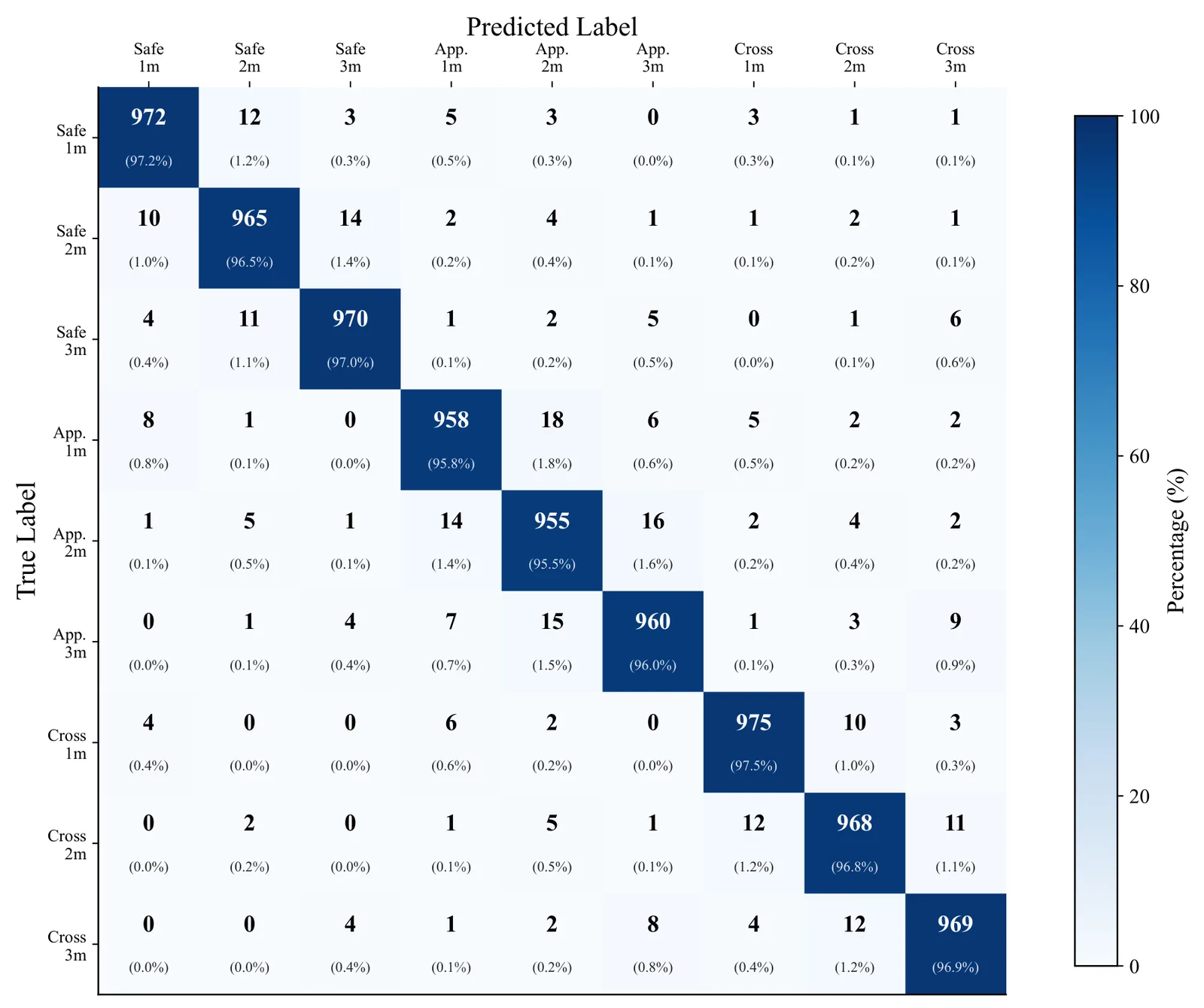
Normalized confusion matrix for motion-state recognition at different sensing distances. The nine categories combine three motion states (Safe, Approach, and Cross) with three sensing distances (1, 2, and 3 m).

The normalized confusion matrix of the proposed method is shown in Figure 17. It should be clarified that the primary detection task remains a binary classification problem, i.e., *crossing* versus *non-crossing*. Figure 17 provides a more detailed analysis by further dividing the test samples according to three motion states (*Safe*, *Approach*, and *Cross*) and three sensing distances (1, 2, and 3 m), resulting in nine state–distance combinations.

The matrix was constructed by comparing the predicted state–distance category with the corresponding ground-truth annotation. Therefore, the nine-class representation is a distance-conditioned diagnostic analysis rather than a redefinition of the final classification task. For the binary evaluation, the three Cross categories are treated as positive samples, whereas the Safe and Approach categories are treated as non-crossing samples. The concentrated diagonal distribution indicates that the proposed method maintains stable state discrimination across different sensing distances with limited confusion between crossing and non-crossing states.

Overall, the experimental results demonstrate that the proposed acoustic sensing framework provides reliable personnel crossing detection in the simulated underground environment and maintains stable discrimination across different sensing distances.

## 6. Discussion

Although the proposed acoustic-based crossing behavior sensing system achieves reliable detection performance under typical underground conditions, several limitations remain and require further investigation.

### 6.1. Limitations in Multi-Target Sensing

The proposed temporal state machine (TSM) and distance–velocity fusion model are mainly designed for single-target crossing behavior with a typical “approach–cross–leave” trajectory. However, practical mining environments may involve multiple workers or complex movements, such as loitering and repeated approaches near the conveyor belt. When multiple targets appear simultaneously, their acoustic echoes may overlap in time and frequency domains, affecting the accuracy of ToF ranging and Doppler velocity estimation, which may further influence state transition decisions. Future work will investigate acoustic array sensing, beamforming, and angle-of-arrival (AoA) estimation techniques to achieve multi-target separation, independent trajectory tracking, and behavior recognition.

### 6.2. Impact of Extreme Mechanical Vibrations

The proposed adaptive feature weighting and continuous-frame confirmation strategies can effectively suppress normal conveyor noise and transient disturbances. However, extreme operating conditions, such as heavy-load startup, strong mechanical vibration, and severe coal impact, may introduce low-frequency structural interference and degrade acoustic feature quality. These disturbances may reduce the reliability of velocity estimation and increase the risk of confusing mechanical motion with human movement. Future studies will explore multimodal fusion with conveyor operating information, such as motor current and load status, as well as lightweight temporal learning models to improve nonlinear interference modeling and vibration robustness.

### 6.3. Practical Deployment and Scalability

The proposed ACBSS system adopts commercial acoustic components and provides advantages including low cost, illumination independence, and dust resistance. However, the current prototype is based on a single sensing node, which limits its scalability for long-distance conveyor monitoring. Future work will focus on distributed acoustic sensing and edge computing architectures, where multiple low-power sensing nodes can be deployed along conveyor lines. By integrating local event detection, underground communication, and conveyor control systems, the proposed framework can be further extended toward a closed-loop safety monitoring system.

### 6.4. Impact of Environmental Temperature and Humidity on Sound Velocity

The proposed ToF-based ranging mechanism inherently relies on the acoustic propagation velocity *c*. In the current laboratory experiments, the ambient temperature and humidity were strictly controlled, allowing the use of a constant sound speed (c=340 m/s). However, in real underground coal mines, significant temperature and humidity gradients exist, which can cause systematic ranging errors if not properly addressed.

The speed of sound in air is primarily affected by temperature *T* (in °C) and can be approximated by the empirical formula:

(27) $$c\left(T\right)\approx 331.3\sqrt{1+\frac{T}{273.15}}\approx 331.3+0.606T\;\left(m/s\right)$$

A temperature variation of ΔT=10 °C would induce a sound speed deviation of approximately Δc≈6 m/s, leading to a relative ranging error of ΔR/R=Δc/c≈1.7%. For a monitoring distance of 3 m, this translates to an absolute error of ∼5.1 cm, which may affect the precise determination of the hazardous boundary $d_{belt}$. Humidity also exerts a minor influence on sound speed, though its effect is secondary to temperature.

To address this limitation in future field deployments, a dynamic sound speed compensation mechanism will be integrated into the system hardware. By deploying low-cost digital temperature and humidity sensors (e.g., SHT3x) near the acoustic transceiver, the real-time environmental sound speed $c_{\mathrm{real}}$ can be calculated and dynamically fed into the ToF estimation module (Equation (13)). This hardware-level compensation will effectively eliminate the systematic ranging bias caused by underground environmental gradients, further enhancing the spatial accuracy of the proposed framework.

## 7. Conclusions

To address the challenges of low illumination and strong mechanical interference in underground conveyor belt monitoring, this paper proposes an acoustic echo-based distance–velocity feature fusion method for unauthorized personnel crossing detection. A spectral-prior-based acoustic interference suppression and multipath mitigation strategy is employed to enhance human echoes, while ToF and micro-Doppler analysis are combined to extract complementary distance and radial velocity features. Based on these features, a distance–velocity coupled temporal discrimination model integrating adaptive feature weighting, a temporal state machine (TSM), and continuous-frame confirmation is developed to suppress false alarms caused by static obstacles, mechanical vibration, and transient disturbances. Experimental results on the laboratory conveyor platform demonstrate an F1-score of 96.2% under the standard 2 m crossing scenario, while an F1-score of 93.5% is obtained in the simulated underground tunnel evaluation. The results further demonstrate the illumination-independent nature of the proposed acoustic sensing framework. Nevertheless, the current system has several limitations that should be addressed before large-scale practical deployment. In particular, the present TSM is primarily designed for single-target crossing, and the simultaneous presence of multiple personnel may produce overlapping acoustic responses in both the time and frequency domains, thereby degrading target separation and distance–velocity estimation. In addition, the current experiments do not fully reproduce extreme underground conditions such as high-concentration coal dust, strong ventilation airflow, and severe temperature–humidity variations. Future work will therefore focus on multi-target separation using acoustic arrays, beamforming, and angle-of-arrival estimation, together with long-term on-site validation under authentic underground operating conditions. These developments are expected to further improve the robustness and practical applicability of the proposed acoustic sensing framework for intelligent conveyor belt safety monitoring.

## Figures and Tables

**Table 1:** Main parameter settings and determination criteria of the proposed ACBSS.

Parameter	Value	Function	Determination Criterion
α	1.0	Wiener regularization	Background-noise calibration
STFT window	256 samples	Doppler estimation	Time–frequency resolution trade-off
STFT overlap	50%	Doppler estimation	Temporal continuity
*Q*	0.001	Kalman process noise	Calibration trajectories
*R*	0.01	Kalman measurement noise	Background measurements
$N_{w}$	10 frames	Feature normalization	Local statistical stability
$d_{\mathrm{belt}}$	1.0 m	Hazard boundary	Conveyor monitoring geometry
$v_{\mathrm{th}}$	0.05 m/s	Motion validation	Background velocity distribution
ΔT	3.0 s	TSM time window	Crossing-duration statistics
*k*	2.0	Adaptive threshold	Background-score statistics
$N_{c}$	3 frames	Alarm confirmation	Transient-disturbance duration

**Table 2:** Key parameters of the proposed FMCW acoustic sensing system.

Parameter	Value/Specification
Sampling frequency ($f_{s}$)	48 kHz
Start frequency ($f_{start}$)	18 kHz
End frequency ($f_{end}$)	22 kHz
Bandwidth (*B*)	4 kHz
Chirp duration ($T_{c}$)	40 ms
Chirp rate (*K*)	$1\times 10^{5}$ Hz/s
Range FFT points ($N_{range}$)	2048
Doppler FFT points ($N_{doppler}$)	256
Chirps per frame	64
Transmitter	Commercial loudspeaker pair
Receiver	Omnidirectional condenser microphone
Sound Pressure Level (SPL)	∼85 dB at 1 m
Monitoring distance	1–4 m
Geometric arrangement	Monostatic with 10 cm TX-RX baseline

**Table 3:** Sensitivity analysis of the main parameters around their nominal settings.

Parameter	Nominal Value	Analyzed Range	Main Influence
$d_{\mathrm{belt}}$	1.0 m	0.8–1.2 m	Spatial decision boundary
$v_{\mathrm{th}}$	0.05 m/s	0.03–0.07 m/s	Clutter rejection/missed motion
ΔT	3.0 s	2.0–4.0 s	State-transition tolerance
$C_{d},C_{v}$	SNR-adaptive	$C_{d}+C_{v}=1$	Distance–velocity contribution

**Table 4:** Performance comparison of different feature combinations under the standard crossing scenario.

Method	Accuracy (%)	Precision (%)	Recall (%)	F1-Score (%)
Distance-only	86.2	78.4	93.6	85.3
Velocity-only	88.4	91.5	81.2	86.0
Proposed	96.5	96.8	95.6	96.2

**Table 5:** Detection performance under different ambient noise levels and conveyor load conditions.

Condition	Noise (dB)	Accuracy (%)	Precision (%)	Recall (%)	F1-Score (%)
Empty-load	∼65	97.2	97.5	96.8	97.1
Medium-load	∼75	96.4	96.8	95.6	96.2
Full-load	∼85	92.0	92.7	90.2	91.4

**Table 6:** Performance comparison of different detection methods in the simulated underground tunnel environment.

Method	Accuracy (%)	Precision (%)	Recall (%)	F1-Score (%)
Low-light vision	79.4	83.2	76.5	79.7
Infrared beam	76.5	68.2	89.4	77.3
**Proposed**	**93.8**	**94.2**	**92.8**	**93.5**

## Data Availability

Dataset available on request from the authors.

## Abbreviations

The following abbreviations are used in this manuscript:

FMCW	Frequency-Modulated Continuous Wave
TOF	Time of Flight
UWB	Ultra-Wideband
DAS	Distributed Acoustic Sensing
ASFF	Adaptively Spatial Feature Fusion
MLFPN	Multi-Level Feature Pyramid Network
POSP	Principle of Stationary Phase
IF	Intermediate Frequency
ACBSS	Acoustic-based Crossing Behavior Sensing System
SNR	Signal-to-Noise Ratio
PSD	Power Spectral Density
STFT	Short-Time Fourier Transform
KF	Kalman Filter
TSM	Temporal State Machine
FFT	Fast Fourier Transform
SPL	Sound Pressure Level
TX	Transmitter
RX	Receiver
RMS	Root Mean Square
RH	Relative Humidity
TP	True Positive
TN	True Negative
FP	False Positive
FN	False Negative
ROC	Receiver Operating Characteristic
PR	Precision–Recall
AUC	Area Under the Curve
AoA	Angle of Arrival
